# Coupling CZE, Liquid‐Phase Ion Mobility, to MS/MS for Quantitative Top‐Down Proteomics: Revealing Significant Proteoform Differences Between Healthy and Alzheimer's Disease Brains

**DOI:** 10.1002/pmic.70041

**Published:** 2025-09-14

**Authors:** Mehrdad Falamarzi Askarani, Fei Fang, Scott E. Counts, Liangliang Sun

**Affiliations:** ^1^ Department of Chemistry Michigan State University East Lansing Michigan USA; ^2^ Department of Translational Neuroscience College of Human Medicine Michigan State University Grand Rapids Michigan USA

**Keywords:** Alzheimer's disease, CZE‐MS/MS, human brain, proteoform, tau phosphorylation, top‐down proteomics

## Abstract

Alzheimer's disease (AD) is a neurodegenerative disorder characterized by cognitive decline and pathological protein aggregation. Comprehensive quantitative proteomics of brain tissues from AD patients is critical for pursuing a better understanding of the molecular mechanisms that drive AD progression. Here, we present one of the first quantitative top‐down proteomics (TDP) studies of postmortem cortex samples from AD patients and healthy controls to profile their proteoform differences by coupling capillary zone electrophoresis (CZE, liquid‐phase ion mobility) to tandem mass spectrometry (MS/MS). We identified 3191 unique proteoforms and uncovered a drastic transformation in the proteoform profile in AD compared to healthy controls. Over 2200 proteoforms were exclusively identified in either AD or healthy control samples, and 157 proteoforms identified in both AD and control samples showed statistically significant abundance differences between the two conditions. Gene Ontology and pathway analysis of the genes associated with those proteoforms revealed broad changes in biological processes in AD brains, for example, telomere organization, substantia nigra development, amyloid fibril formation, microtubule cytoskeleton organization, progressive neurological disorders, long‐term synaptic potentiation, and axogenesis. These biological processes are highly associated with the development of AD. Our study revealed a pool of potential novel proteoform biomarkers of AD in human brain samples for early diagnosis and therapy development.

**Summary:**

- Alzheimer's disease (AD) is a chronic neurodegenerative disease, destroying brain cells and causing thinking ability and memory to decline over time. Proteins (e.g., amyloid and tau) play key roles in the development of AD.
- Global and accurate protein measurement of human brains of AD patients and healthy controls will shed new light on the molecular mechanisms driving AD progression and discover new biomarkers for AD diagnosis and therapeutic development.
- Here, we performed the first CZE‐MS/MS‐based quantitative top‐down proteomics (TDP) of a small cohort of AD human brain samples and healthy controls (5 AD and 5 control) to determine the differentially quantified proteoforms between the two health conditions.
- Over 3000 proteoforms were identified, and only about 700 proteoforms were detected in both conditions, indicating drastically different proteoform profiles between the two conditions.
- The differentially quantified proteoforms (e.g., tau, neurogranin, and calmodulin‐1 proteoforms) are associated with biological processes relevant to AD development, for example, amyloid fibril formation, microtubule disruption, synaptic transmission, and axogenesis.
- The results offer a deep view of the proteoform transformation in the AD human brain compared to the healthy control, providing potential proteoform biomarkers for AD diagnosis and proteoform targets for therapeutic development.

**Abbreviations**
BGE: background electrolyte
CZE: capillary zone electrophoresis
HCD: higher‐energy collisional dissociation
MS: mass spectrometry
MS/MS: tandem mass spectrometry
NCE: normalized collision energy
TDP: top‐down proteomics

## 1 Introduction

Alzheimer's disease (AD) represents the most prevalent neurodegenerative condition and stands as the primary cause of dementia among elderly populations [1, 2]. Individuals suffering from AD undergo a progressive deterioration of cognitive abilities, such as memory retention and reasoning capacity, alongside observable alterations in their social behaviors and interpersonal interactions, which increasingly compromise their capacity for independent living [3, 4]. Two particular proteins are hallmarks of AD neuropathology, amyloid beta (Aβ), and the hyperphosphorylated form of microtubule‐associated protein tau (tau), both of which demonstrate robust correlation with disease progression and manifestation [5, 6, 7, 8]. These phenomena suggest a potentially substantial change in the human brain proteome during AD progression. Therefore, we expect that comprehensive and accurate measurement of protein differences in the brains of healthy controls and AD patients will offer a bird's‐eye view of the proteome dynamics of AD and provide a better understanding of pathogenic molecular mechanisms of disease progression.

Mass spectrometry (MS)‐based bottom‐up proteomics (BUP) has been widely used to study the brain proteome dynamics in AD for new biomarker discovery [8, 9, 10, 11, 12, 13, 14]. However, due to the enzymatic digestion of proteins in BUP, it fails to characterize proteoforms, representing all forms of protein molecules from the same gene due to genetic alleles, alternative splicing, proteolytic processing, and post‐translational modifications (PTMs) [15, 16]. Proteoforms from the same gene can have divergent biological functions [17, 18, 19, 20, 21, 22, 23, 24, 25]. It is essential to characterize the brain proteome in a proteoform‐specific manner to achieve an accurate understanding of brain function and AD progression. MS‐based top‐down proteomics (TDP) is ideal for this purpose since it enables analysis of intact proteoforms, enhancing detection of pathology‐associated proteoforms and strengthening genotype‐phenotype correlations [26, 27]. Unfortunately, very few MS‐based TDP studies have been published to delineate the proteoform‐level changes in the brain during AD progression for the discovery of proteoform biomarkers [28, 29, 30, 31]. For example, Fulcher et al. performed a large‐scale quantitative TDP study of AD human brain samples and identified more than 11,000 proteoforms from 103 brain samples [28]. They discovered strong correlations between Aβ proteoforms and amyloid plaques, global cognitive function, or cerebral amyloid angiopathy.

Capillary electrophoresis (CE)‐MS has been well‐documented as a powerful alternative to the commonly used reversed‐phase liquid chromatography (RPLC)‐MS for TDP due to its high separation efficiency and high detection sensitivity for proteoforms [32, 33, 34, 35, 36, 37, 38]. CE‐MS‐based TDP has also been used to study brain samples [35, 39, 40] and tau proteoforms [41] by our group recently. Capillary zone electrophoresis (CZE), the simplest form of CE, separates analytes in a time scale of seconds to minutes based on their mobility in the liquid phase under a strong electric field, and the mobilities correlate to their mass‐to‐charge ratios. CZE is similar to ion mobility separation, which separates analytes in the gas phase in a time scale of milliseconds under an electric field according to their mobility, strongly influenced by the size and charge of the analytes [42]. Therefore, CZE is also recognized as a liquid‐phase ion mobility separation. Here, for the first time, we utilized CZE‐tandem mass spectrometry (MS/MS)‐based TDP, following size exclusion chromatography (SEC) fractionation, for the quantitative measurement of proteoforms in temporal cortex obtained postmortem from AD subjects and age‐matched healthy controls. Our analytical approach identified 3191 unique proteoforms spanning 500 distinct proteins, including many proteoforms showing statistically significant abundance differences between the two biological conditions.

## 2 Materials and Methods

### 2.1 Materials and Reagents

Ammonium bicarbonate (ABC), dithiothreitol (DTT), and Amicon Ultra centrifugal filter units (0.5 mL, 10 kDa molecular weight cutoff [MWCO]) were obtained from Sigma–Aldrich (St. Louis, MO). LC/MS grade water, LC‐grade acetic acid (AA), and methanol (MeOH) were bought from Fisher Scientific (Pittsburgh, PA). Bare fused silica capillaries (50 µm inner diameter, 360 µm outer diameter) were purchased from Polymicro Technologies. Acrylamide was procured from Acros Organics (Fair Lawn, NJ). Complete mini protease inhibitor cocktail and PhosSTOP (EASYpacks) were acquired from Roche (Indianapolis, IN).

### 2.2 Sample Preparation

Frozen inferior temporal cortex (Brodmann area 20) tissue blocks were obtained postmortem from control and mild AD subjects (*n* = 5/group) enrolled in the Michigan Alzheimer's Disease Research Center (MADRC) clinical cohort [43]. These subjects underwent longitudinal structured neuropsychological and clinical evaluations in accordance with the National Alzheimer's Coordinating Center Uniform Dataset 2.0 [44] and routine neuropathological diagnostic analysis, including Braak staging [43, 44, 45]. Each subject's final diagnosis included their last antemortem Mini‐Mental State Exam (MMSE) score as a measure of global cognitive function prior to death [46, 47]. Exclusion criteria included evidence of comorbid neurodegenerative or neurological diseases such as parkinsonism or large strokes. The entire protocol related to the collection of human brain samples was performed in accordance with guidelines defined by the Institutional Review Board of Michigan State University. Approximately ∼300 mg of frozen tissue was excised from each block under sterile conditions to mitigate external protein contamination. Tissue specimens of 50 mg each were separated from healthy controls and mild AD subjects for downstream processing. The tissues were transferred to a 15 mL conical tube and resuspended in 1 mL of lysis buffer (8 M urea, 100 mM ABC [pH 8.0], supplemented with “complete” protease inhibitor mixture and “PhosSTOP” phosphatase inhibitors). The tissues were homogenized using a Fisher Scientific Homogenizer 150 and sonicated on ice with a VWR Scientific Branson Sonifier 250 to enhance protein extraction and then clarified by centrifugation (18,000 × *g*, 10 min, 15°C). Protein concentration in the supernatant was measured using a bicinchoninic acid (BCA) assay (Fisher Scientific, Pittsburgh, PA). Before MS analysis, the protein samples were reduced with 5 mM DTT at 37°C for 30 min. A buffer exchange was carried out using a Microcon‐10 kDa centrifugal filter (Sigma–Aldrich) at 14,000 × *g* for 10 min, followed by three washes with 10 mM ammonium acetate (pH 6.9). The proteoform sample on each membrane was redissolved in 30–40 µL of 10 mM ammonium acetate (pH 6.9) and subjected to the SEC fractionation.

### 2.3 SEC Fractionation

Protein fractionation was performed by SEC using an Agilent 1260 Infinity II HPLC system with an SEC column (Agilent, dimensions: 4.6 × 300 mm, 5 µm particle size, 500 Å pore size). An ultraviolet‐visible detector monitored separation at 254 nm wavelength.

For the experiment, 450 µg of protein from each sample (five AD patients and five healthy controls) was injected onto the column. Separation was performed using a 10 mM ammonium acetate buffer (pH 6.9) at a flow rate of 0.25 mL/min. Collection proceeded in 3‐min intervals, yielding three fractions per sample (750 µL each). All fractions underwent lyophilization via speed vacuum and were subsequently reconcentrated to 30 µL in 10 mM ammonium acetate buffer (pH 6.9).

### 2.4 CZE‐MS/MS Analysis

The CZE‐MS/MS setup integrated a Beckman Coulter CESI 8000 Plus CE system with a Thermo Fisher Scientific Orbitrap Exploris 480 mass spectrometer, employing an in‐house designed electrokinetically driven sheath‐flow CE‐MS nanospray interface [48, 49]. A 1‐m‐long fused silica capillary (50 µm i.d./360 µm o.d.) was coated with linear polyacrylamide (LPA) according to our previous procedure [50]. The capillary's outer diameter at one end was tapered to 70–80 µm using hydrofluoric acid etching [51]. Approximately 112 nL of each sample was pressure‐injected at a 5‐psi pressure for 19 s, in accordance with Poiseuille's law. Separation was performed by applying 20 kV at the injection end for 80 min, while a 2 kV voltage was used at the CE‐MS interface for electrospray ionization (ESI). Between runs, the capillary was flushed for 10 min at 50 psi using the background electrolyte (BGE). The ESI emitter was fabricated from a glass capillary (0.75 mm i.d./1.0 mm o.d., 10 cm length) using a Sutter P‐1000 micropipette puller, with an orifice diameter of 20–40 µm. The CZE BGE consisted of 5% (v/v) AA (pH 2.4), while the ESI sheath buffer contained 0.2% (v/v) formic acid and 10% (v/v) methanol.

All experiments were performed on a Thermo Fisher Scientific Orbitrap Exploris 480 mass spectrometer operating in data‐dependent acquisition (DDA) mode. The MS1 scans were acquired with a resolution of 120,000 (at *m*/*z* 200), a single microscan, and a scan range of 600–2000 *m*/*z*. The automatic gain control (AGC) target was set to 300%, and the maximum injection time was adjusted automatically. Precursor ions were isolated with a 2 *m*/*z* window and fragmented via higher‐energy collisional dissociation (HCD) at a 25% normalized collision energy (NCE). Only ions with intensities exceeding 10,000 and charge states between 5+ and 60+ were selected for fragmentation. MS/MS spectra were recorded at a resolution of 60,000 (at *m*/*z* 200) with three microscans, an AGC target of 100%, and a maximum of six dependent scans. Dynamic exclusion was enabled for 30 s with a 10 ppm mass tolerance, and the “Exclude isotopes” option was activated to minimize redundant fragmentation.

### 2.5 Data Analysis

For TDP analysis, the complex sample data were processed using Xcalibur software (Thermo Fisher Scientific, v4.5) to extract proteoform intensities and migration times. Electropherograms were exported from Xcalibur and refined using Inkscape (v1.3.2) for final figure generation.

Proteoform identification and quantification in AD and healthy control samples were performed using the TopPIC pipeline (top‐down proteoform identification and characterization) [52]. The workflow began with converting RAW files to mzML format via MSConvert [53]. The process of converting precursor and fragment isotope clusters into monoisotopic masses and proteoform features was carried out using TopFD (Top‐down MS Feature Detection, version 1.7.8) [54]. The mass spectra and proteoform feature information were saved in msalign and text files, respectively. The database search was conducted via TopPIC (version 1.7.8). The analysis permitted a maximum of one unexpected mass shift, with mass error tolerances set to 10 ppm for both precursors and fragments. Unknown mass shifts were restricted to ≤500 Da. To assess identification confidence, false discovery rates (FDRs) were calculated using a target‐decoy approach. Identified proteoforms were filtered at 1% FDR thresholds for both proteoform‐spectrum matches (PrSMs) and proteoform‐level identifications [55, 56]. In addition to obtaining individual TopPIC outputs for each fraction, a combined output from all SEC fractions per sample was also obtained. The PrSM cluster error tolerance was set to the default value of 1.2 Da to reduce proteoform redundancy.

For label‐free quantification (LFQ) of proteoforms across human brain samples, we employed TopDiff (v1.7.8) to determine the LFQ intensities of proteoforms across all the samples [36]. The analysis was conducted using the software's default parameters. The TopDiff software is part of the TopPIC suite package (https://www.toppic.org/software/toppic/index.html). To ensure robustness of our quantification results, we performed the analysis by using three different mass tolerance parameters (1.2, 2.2, and 3.2 Da) in TopDiff, showing consistent results across all settings. To determine the differentially quantified proteoforms between healthy controls and AD samples, the quantified proteoforms were further processed by the Student's *t* test using the Perseus software [57].

## 3 Results and Discussions

**FIGURE 1:**
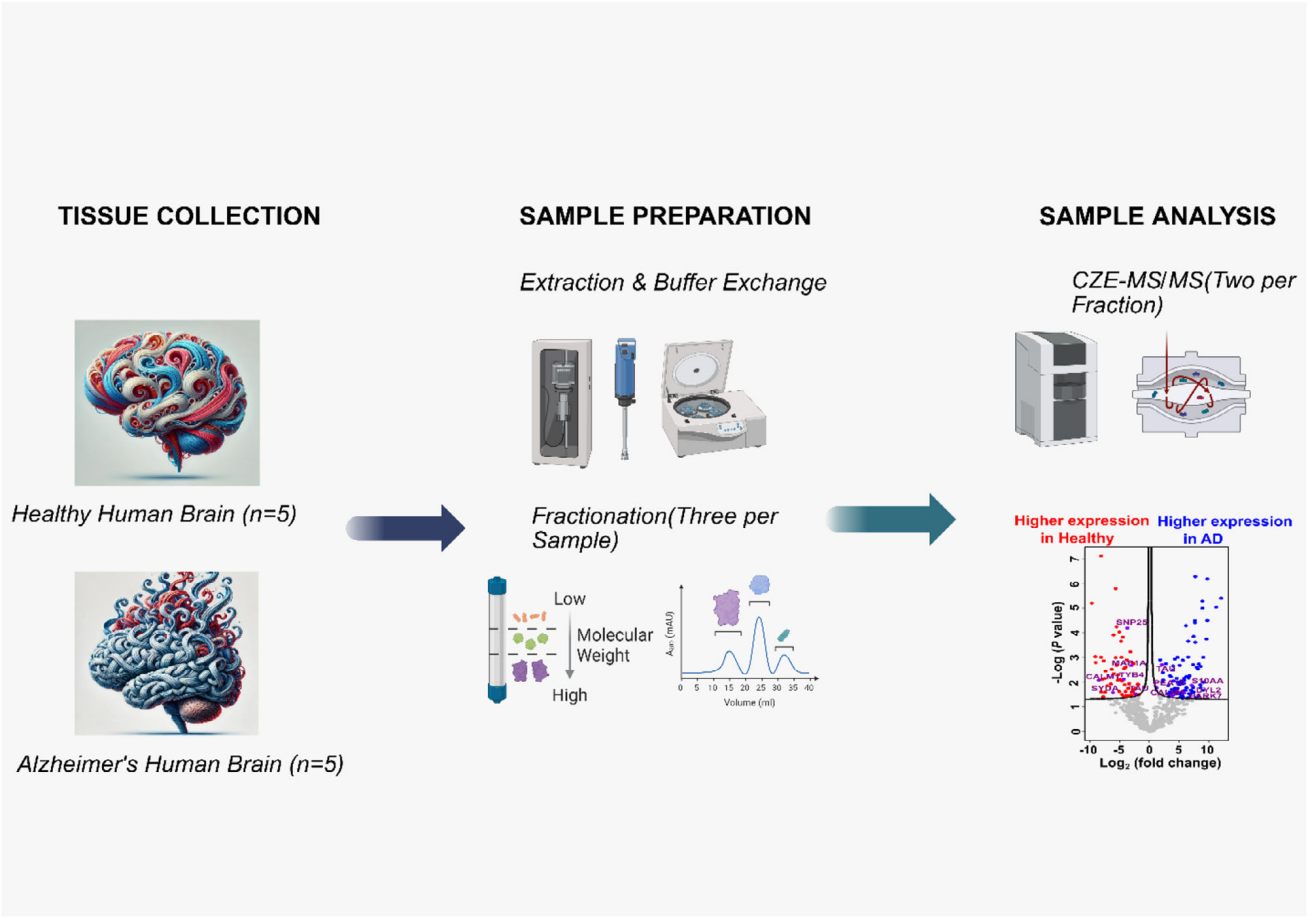
Workflow for TDP analysis of 10 human brain samples (5 Alzheimer's disease vs. 5 healthy controls). Human brain proteoforms were extracted from each tissue sample and separated by size‐exclusion chromatography (SEC), generating 3 fractions per sample. After that, the 30 SEC fractions (3 fractions × 5 AD + 3 fractions × 5 controls) were analyzed by CZE‐MS/MS for proteoform identification and label‐free quantification (LFQ), and each fraction was measured in technical duplicate, resulting in 60 CZE‐MS/MS runs. The figure was made using BioRender. AD, Alzheimer's disease; CZE, capillary zone electrophoresis; MS/MS, tandem mass spectrometry; TDP, top‐down proteomics.

To determine the differentially quantified proteoforms between AD and healthy controls, in this pilot study, we analyzed temporal cortex from a small cohort of control and AD subjects using a two‐dimensional (2D) SEC‐CZE‐MS/MS approach based on our previous works [20] Figure 1. The workflow began with protein extraction through tissue homogenization and sonication in a lysis buffer, followed by buffer exchange using a centrifugal filter device with a 10‐kDa MWCO. To enhance proteome coverage and reduce sample complexity, we performed SEC fractionation, yielding three distinct fractions per sample based on molecular weight distribution. The fractionated proteoform mixtures were then analyzed by dynamic pH junction‐based CZE‐MS/MS [37]. Each SEC fraction was analyzed in technical duplicate to validate measurement reproducibility, resulting in a total of 60 CZE‐MS/MS runs across all samples (5 AD cases × 3 SEC fractions × 2 replicates + 5 controls × 3 SEC fractions × 2 replicates). Subsequently, the combined output from all SEC fractions per sample was obtained. As shown in Figure S1, the combined data from one representative AD sample (AD2, Figure S1A) and healthy control (H1, Figure S1B) show the identification of 438 and 693 proteoforms, respectively. The total number of proteoform identifications per sample is substantially higher than that of each SEC fraction, indicating the effectiveness of SEC fractionation for improving the proteome coverage. Figure S2 shows the violin plots of mass distributions of identified proteoforms from CZE‐MS/MS across three SEC fractions from an AD sample (AD2, Figure S2A) and a healthy sample (H1, Figure S2B). The median proteoform mass gradually decreases from Fraction 1 to Fraction 3 in both conditions. For example, AD samples show a decrease from SEC‐1 (∼13 kDa) to SEC‐3 (∼7 kDa), while healthy samples demonstrate a similar trend from SEC‐1 (∼10 kDa) to SEC‐3 (∼6 kDa). This progressive mass reduction across fractions indicates effective size‐based separation by SEC, with technical replicates showing consistent mass distributions within each fraction. The results demonstrate that SEC‐CZE‐MS/MS successfully improves the identification of proteoforms across a broad molecular weight range.

We further analyzed proteoform intensity correlations between SEC fractions in one AD (AD2) and one healthy control (H1) sample, Figure S3. Technical replicates of the same fraction show strong linear correlations in proteoform intensity (AD2: Pearson *r* = 0.89–0.95; H1: *r* = 0.94–0.98), while inter‐fraction correlations are substantially weaker (AD2: *r* = 0.53–0.80; H1: *r* = 0.15–0.85). The data indicate that CZE‐MS/MS analysis is reproducible in terms of proteoform intensity based on the replicate data, and SEC separation of proteoforms is effective. We further analyzed the proteoform intensity correlations between any two biological replicates of ADs and healthy controls, Figure S4. The Pearson correlation coefficient (*r*) ranges from 0.73 to 0.85 for AD samples (Figure S4A) and from 0.82 to 0.85 for healthy control samples (Figure S4B). The data show that biological replicates of healthy controls and ADs have lower linear correlation coefficients than the technical replicates of the same brain sample (Figure S3), suggesting significant biological heterogeneity between brain samples.

Subject demographic, clinical, and neuropathological characteristics are shown in Table S1. The control and AD subjects did not display significant differences in age at death, education, and postmortem interval. However, AD subjects showed significantly lower antemortem MMSE scores and significantly higher postmortem Braak stage pathology compared to control subjects.

### 3.1 Distinct Proteoform Profiles Discriminated AD From Healthy Brains

**FIGURE 2:**
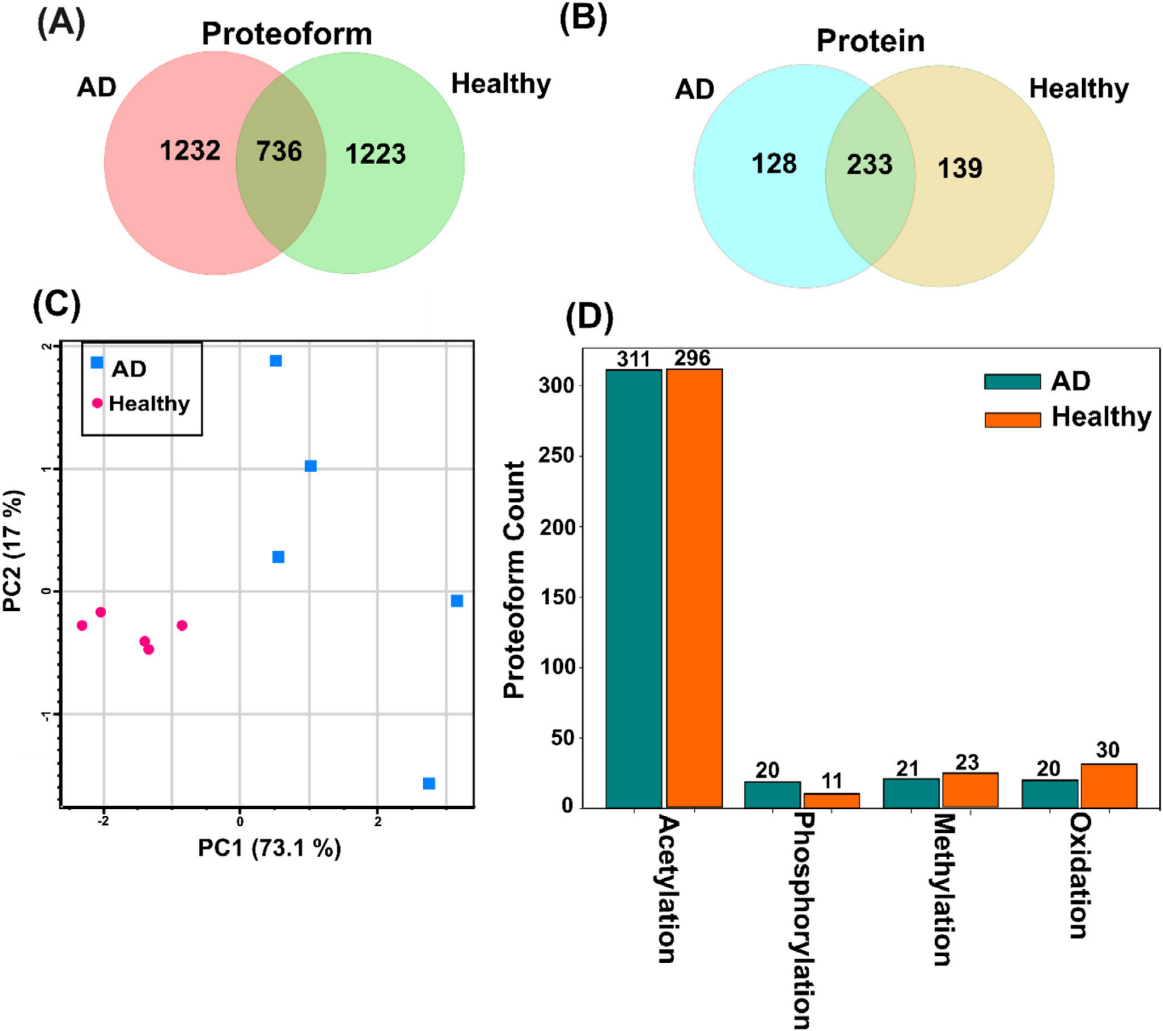
Summary of the proteoform profile data of healthy controls and Alzheimer's diseases (ADs). (A) Venn diagram showing overlap between AD and healthy samples for identified proteoforms. (B) Venn diagram showing overlap between AD and healthy samples for identified proteins. (C) Principal component analysis (PCA) separates the controls and ADs well based on the overlapped proteoforms. (D) Distributions of common post‐translational modifications (PTMs, e.g., acetylation, phosphorylation, methylation, and oxidation) identified in the proteoforms from AD and control.

We identified 3191 unique proteoforms of 500 proteins from control and AD brain samples. The identified and quantified proteoforms are listed in Supporting Information SII. The two conditions have substantial differences in the proteoform profiles. First, only 736 out of 3191 proteoforms (23%) were identified in both AD and control, Figure 2A. A total of 1232 proteoforms were unique to AD samples, and 1223 proteoforms were unique to healthy controls. Figure 2B shows the protein‐level overlap between the AD and control samples, and 233 out of 500 proteins (∼47%) were identified in both conditions. Second, the AD and control samples can be well separated by the multivariate principal component analysis (PCA) using the intensity data of overlapped proteoforms, Figure 2C. The five control samples were grouped closely together, whereas the AD samples had more diverse proteoform intensity data, indicating substantially higher proteoform heterogeneity within the AD samples.

We further highlighted this heterogeneity using several principal proteoforms, Figure S5. The relative standard deviations (RSDs) of proteoform intensity are much higher in ADs compared to healthy controls (H) for those five principal proteoforms driving the separations in the PCA analysis. These proteoforms include one N‐terminal acetylated thymosin beta‐4 (TMSB4X) proteoform carrying a −17.9919‐Da mass shift (A), one truncated neurogranin (NRGN) proteoform (B), one polyubiquitin‐C (UBC) proteoform carrying a −99.0225‐Da mass shift (C), one N‐terminal acetylated fully length beta‐synuclein proteoform carrying a +14.3677‐Da mass shift (SNCB) (D), and one N‐terminal acetylated thymosin beta‐10 (TMSB10), Figure S5. Importantly, those proteins have been highly relevant to the pathogenesis of AD according to the literature. The data here further highlights the complexity of AD pathogenesis, which involves the dysregulation of multiple cellular and molecular functional pathways.

We further analyzed the amyloid precursor protein (APP) proteoforms between AD and healthy controls, considering their importance in the pathogenesis of AD. In total, we identified eight APP proteoforms in AD samples and nine in healthy controls with detectable signal in at least one sample, as listed in Supporting Information SII. In total, 13 APP proteoforms were identified, and 4 of them were shared by ADs and controls. Those APP proteoforms include amyloid beta 42 (Aβ42), Aβ40, modified (e.g., oxidation) and/or N‐terminally truncated Aβ42 proteoforms. As expected, Aβ42 and Aβ40 were much higher abundance in ADs compared to controls (4.8E8 ± 8.7E8 vs. 8.8E6 ± 5.7E6 for Aβ42; 3.1E8 vs. 1.7E6 for Aβ40), thus supporting methodological rigor. Interestingly, the APP proteoforms also show high intensity variations within the AD samples. For example, the Aβ42 proteoform has a 10‐times higher intensity in one AD sample compared to the other four AD samples (2E9 vs. ≤2E8). The findings here align well with the amyloid cascade hypothesis while highlighting unprecedented molecular heterogeneity in AD [58]. The observed PTMs (e.g., methionine oxidation) and proteolytic fragments implicate oxidative stress and disrupted proteostasis in the AD progression [59]. The proteoform‐resolved approach identifies diverse amyloid beta proteoforms resulting from cleavages, offering potential novel insights into the Aβ‐central hypothesis of AD pathogenesis. The data also underscores the value of TDP in neurodegenerative disease research. Moreover, some examples of base peak electropherograms of CZE‐MS/MS are shown in Figure S6, indicating the reproducible measurement of the samples and ensuring the high reliability of our TDP data. The results here fully demonstrate that the proteoform profile of the human brain has a drastic transformation from healthy aged control to AD conditions.

We then studied PTMs on the identified proteoforms in AD and control brain samples. We focused on four common PTMs detected in our TDP datasets, including acetylation, phosphorylation, methylation, and oxidation, Figure 2D. N‐terminal acetylation influences protein stability, folding dynamics, interaction capabilities, and subcellular localization patterns [60]. Phosphorylation events are well‐established regulators of cellular signaling cascades, gene expression mechanisms, and differentiation processes [61]. Methylation modifications play crucial roles in transcriptional regulation networks [62]. We identified 311 proteoforms with acetylation (mass shift +42 Da) in AD versus 296 in healthy controls. We identified 20 proteoforms with phosphorylation (mass shifts of +80 Da for single phosphorylation or +160 Da for double phosphorylation) in AD and 11 phosphorylated proteoforms in healthy controls. Thirty and 20 proteoforms with oxidation (+16‐Da mass shift) were identified in control and AD samples, respectively. Twenty‐one and 23 methylated proteoforms (+14‐Da mass shift) were identified in AD and control, respectively. Furthermore, our TDP approach successfully identified proteoforms with combinations of multiple PTMs. Figure S7A shows the sequences and fragmentation pattern of one beta‐synuclein (SNCB) proteoform containing N‐terminal acetylation as well as phosphorylation and oxidation in the highlighted region (S118‐E131) close to the C‐terminus. SNCB is a critical protein in inhibiting alpha‐synuclein aggregation, which is associated with Parkinson's disease [63] and often occurs in late‐stage AD [64]. Recent studies also suggested SNCB as a blood biomarker of AD [65]. The S118 phosphorylation of SNCB could play critical roles in Parkinson's disease and related diseases, according to the data of alpha‐synuclein [66]. Figure S7B shows one Cofilin‐1 (CFL1) proteoform containing N‐terminal acetylation and mass shifts of 14.025 and 15.999 Da, likely corresponding to methylation and oxidation modifications, respectively, in the middle‐highlighted regions of the sequence. Both SNCB and CFL1 have established roles in AD pathophysiology [63, 65, 66, 67, 68], though the specific contributions of these precisely modified proteoforms to disease progression remain to be elucidated. The ability to detect and quantify these specific proteoforms with PTM combinations established the foundation for further studying their functions.

### 3.2 Quantitative TDP Determined Differentially Quantified Proteoforms Between Healthy and AD Human Brains

**FIGURE 3:**
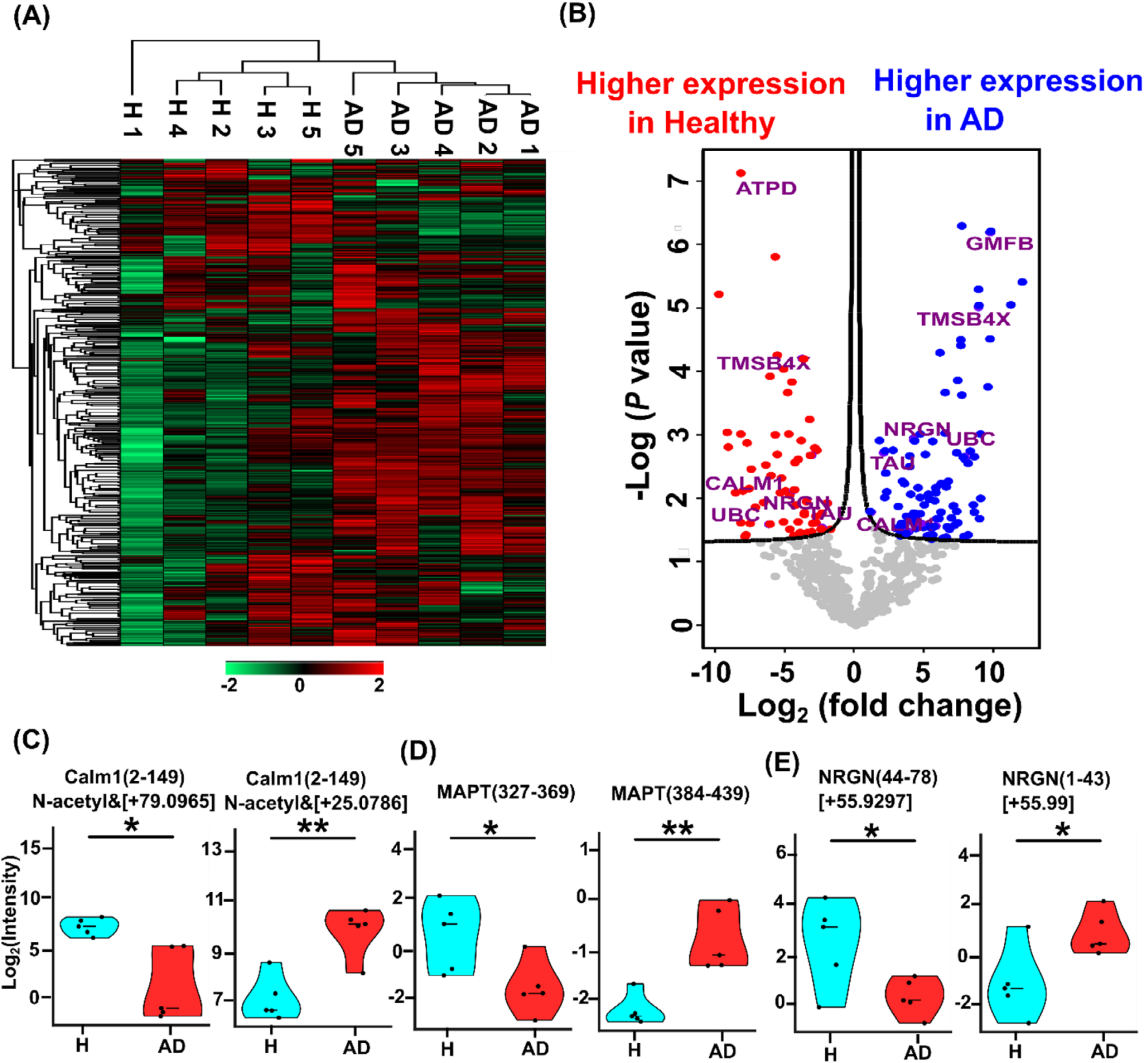
Summary of label‐free quantification (LFQ) data comparing AD and healthy control. (A) Cluster analysis heatmap displaying quantified proteoform (597) LFQ intensity profiles. Data underwent z‐score normalization, with red indicating high abundance and green representing low abundance. (B) Volcano plot visualization of quantified proteoforms (597) determined the higher abundance proteoforms in AD (blue) and in healthy control (red). Some selected differential proteoforms are labeled with their corresponding gene names. (A) and (B) were generated using the Perseus software [1]. The parameters for the volcano plot were set to S0 0.1 and FDR 0.1. Violin plots illustrating abundance difference of two Calm1 proteoforms (C), two MAPT proteoforms (D), and two NRGN proteoforms (E) between AD and healthy control (H). ** *p* < 0.01, * *p* < 0.05. AD, Alzheimer's disease; FDR, false discovery rate; MAPT, microtubule‐associated protein tau 2N4R; NRGN, neurogranin.

Hierarchical clustering analysis of the quantified proteoforms with detectable intensities in every sample (597 proteoforms in total) revealed distinct proteoform intensity profiles in AD and control, clearly separating the AD and control samples into two clusters, Figure 3A. Based on the volcano plot in Figure 3B, 97 proteoforms from 56 proteins exhibited higher abundance in AD samples, and 60 proteoforms from 27 proteins showed elevated expression in control samples. Some example differential proteoforms (e.g., GMFB and ATPD) show the most significant expression differences between AD and control, Figure 3B. The marked glia maturation factor beta (GMFB) full‐length proteoform (∼16614 Da, N‐terminal M removal and N‐terminal acetylation) exhibits drastically higher expression in AD compared to control. GMFB serves as a crucial neurodevelopmental regulator, influencing both glial and neuronal differentiation pathways, and has been suggested as a therapeutic target and biomarker of AD, showing high expression in AD brains [69]. The results indicate that the specific GMFB proteoform quantified here could be a valuable proteoform biomarker of AD. Mitochondrial ATP synthase subunit delta (ATPD) has six proteoforms with obviously higher abundance in the control compared to AD. ATPD has been suggested as a target of oxidative stress in AD [70], and impaired ATP synthesis could contribute to faltering mitochondrial function and cellular energy during disease progression [64]. Those ATPD proteoforms feature an N‐terminal transit peptide (1–22) cleavage and various PTMs (e.g., phosphorylation and methylation).

An interesting finding from this differential analysis was the evidence of bidirectional proteoform regulation for five genes: CALM1, microtubule‐associated protein tau 2N4R (MAPT), NRGN, TMSB4X, and UBC. These genes are associated with the progression of AD, and we observed some proteoforms with higher expression in AD subjects and some proteoforms with higher expression in healthy controls. For example, two and five CALM1 proteoforms have higher expression in AD and control, respectively. Three and nine TMSB4X proteoforms exhibit higher abundance in AD and control samples, respectively. The data highlights the value of proteoform‐specific measurement using MS‐based TDP.

**FIGURE 4:**
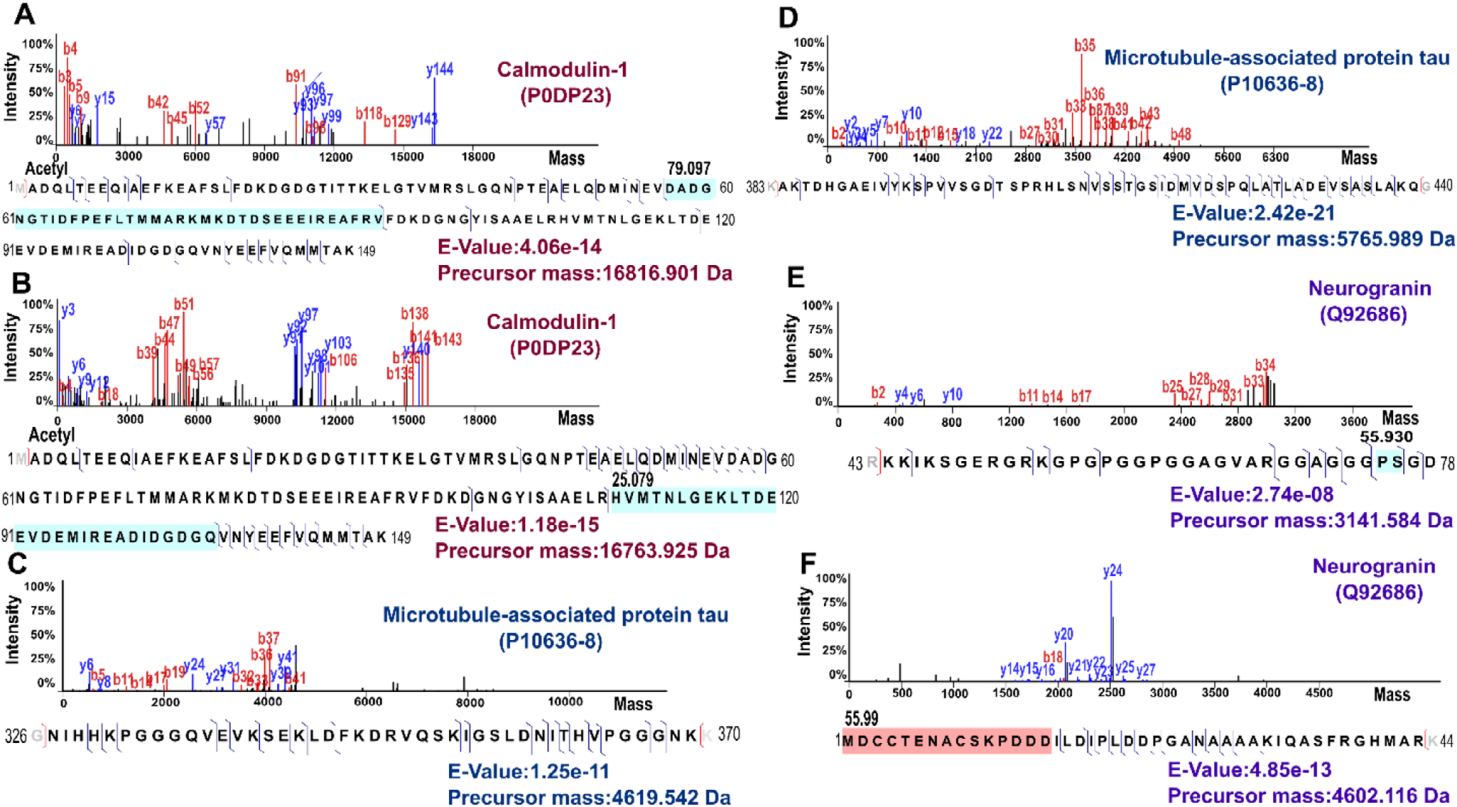
Annotated MS/MS spectra and fragmentation patterns of the differentially expressed proteoforms shown in Figure 3C–E. Calmodulin‐1 (P0DP23) proteoforms with N‐terminal acetylation and a 79‐Da mass shift (A) and with N‐terminal acetylation and one additional 25‐Da mass shift (B). (C) Microtubule‐associated protein tau 2N4R (P10636‐8) proteoform covering amino acid residues 327–369. (D) Microtubule‐associated protein tau (P10636‐8) proteoform covering amino acid residues 384–439. Neurogranin (Q92686) proteoforms with one 56‐Da mass shift covering residues 44–78 (E) and residues 1–43 (F). The proteoform masses and *E* values are labeled. The highlighted regions on the proteoform sequences carry the specific mass shifts or PTMs. The matched b and y fragment ions of proteoforms are labeled on the MS/MS spectra. MS/MS, tandem mass spectrometry; PTM, post‐translational modification.

We then studied several differentially quantified proteoforms of three critical proteins (calmodulin, MAPT, and NRGN) related to AD progression. Calmodulin is a critical calcium‐sensing protein that regulates numerous cellular processes, and its dysregulation is implicated in the amyloid pathway and tangle formation in AD [71]. Our data revealed a striking example of bidirectional regulation for this protein. As evidenced in Figure 3C, we identified two distinct calmodulin‐1 (CALM1) proteoforms with opposite expression patterns. The annotated MS/MS spectra, sequences, and fragmentation patterns are shown in Figure 4A, B. The fragmentation patterns in Figure 4 are all based on single MS/MS scans, not on averaged MS/MS spectra. A CALM1 proteoform with N‐terminal acetylation and one additional 79‐Da mass shift (precursor mass: 16,816.901 Da, *E* value: 4.06e‐14) showed significantly higher abundance in control samples (*p* = 0.008), Figure 4A, while the other CALM1 proteoform with N‐terminal acetylation and a 25‐Da mass shift (precursor mass: 16,763.925 Da, *E* value: 1.18e‐15) was significantly elevated in AD samples (*p* = 0.002), Figure 4B. MAPT is one of the most well‐recognized proteins in AD, and tau protein aggregation into tangles within neurons is one of the main phenotypes of AD. We determined two differentially expressed truncated MAPT proteoforms with opposite expression patterns between AD and healthy control cases (H), Figure 3D. The annotated MS/MS and fragmentation patterns are shown in Figure 4C, D. One truncated MAPT proteoform encompassing amino acid residues 327–369 (precursor mass: 4619.542 Da, *E* value: 1.25e‐11) exhibited significantly higher abundance in control samples (*p* = 0.03), Figure 4C, whereas another MAPT proteoform encompassing amino acid residues 384–439 (precursor mass: 5765.989 Da, *E* value: 2.42e‐21) showed significantly elevated levels in ADs (*p* = 0.002), Figure 4D. NRGN plays a crucial role in synaptic plasticity by sequestering calmodulin in dendritic spines, thereby regulating the calcium/calmodulin‐dependent protein kinase II (CaMKII) signaling cascade, which is essential for long‐term potentiation (LTP) and memory formation [72]. In AD, the cleavage of NRGN disrupts this finely tuned calcium signaling system, leading to impaired synaptic strengthening and cognitive deficits [73]. Figure 3E shows two differentially quantified truncated NRGN proteoforms and one proteoform spanning residues 44–78 with a +55.9297‐Da mass shift (precursor mass: 3141.584 Da, *E* value: 2.74e‐08) had significantly higher abundance in control samples, while the other NRGN proteoform encompassing residues 1–43 with a mass shift of +55.99‐Da could plausibly correspond to a combination of acetylation and methylation modifications, (precursor mass: 4602.116 Da, *E* value: 4.85e‐13) showed significantly higher expression in AD samples. The MS/MS data and fragmentation patterns further indicate the high confidence of those proteoforms, Figure 4E, F.

Tau regulates microtubule stability in healthy neurons, but in AD, abnormal phosphorylation of tau at specific residues disrupts this function, leading to tau detachment from microtubules, misfolding, and aggregation into toxic oligomers and fibrils, and eventually tangles [74]. The accumulation of hyperphosphorylated tau correlates with disease progression, synaptic dysfunction, and neurodegeneration like AD [75]. In this study, we identified eight phosphorylated tau proteoforms from AD and control brain samples, and three of them were identified in both conditions. The proteoforms are listed in Supporting Information SII. Interestingly, we identified five phosphorylated tau (2N4R) proteoforms that are unique to AD (4) or healthy control (1), since those five proteoforms only have detectable signals in either AD or control.

Considering the importance of tau phosphorylation in modulating AD progression, we expect that those five tau proteoforms could be novel proteoform biomarkers of AD. Figure S8 shows two examples of phosphorylated tau (2N4R) proteoforms identified in both AD and control samples regarding annotated MS/MS spectra and fragmentation patterns. Although limited fragment ions prevented exact localization of phosphorylation sites, the observed mass shifts in b‐ions suggested potential phosphorylation sites. For the 2N4R proteoform in Figure S8A, a mass shift of approximately +80 Da in the b25 ion (monoisotopic mass: 2725.3436 Da) indicated a single phosphorylation likely occurring between Thr721 and Leu726, with a higher probability at Thr721 and Ser722. In the proteoform shown in Figure S8B, a mass shift of around +241 Da in the b35 ion (monoisotopic mass: 4033.136 Da) suggested hyperphosphorylation (three phosphorylation sites) within the region spanning Val716 to Val729, most likely at Ser718, Thr721, Ser722, and Ser727. The fragment ions with 98,160, and 18‐Da neutral losses further confirm the phosphorylation modification on the two 2N4R proteoforms.

### 3.3 Gene Ontology (GO) and Pathway Analysis Revealed Broad Biological Process Changes in AD

To understand the collective biological impact of these widespread proteoform changes, we performed GO analysis using the DAVID tool [76] and pathway enrichment analysis. Proteoforms that were upregulated or uniquely detected in AD (Table S2) were significantly associated with various biological processes, such as telomere organization, substantia nigra development, amyloid fibril formation, microtubule cytoskeleton organization, regulation of neuron apoptotic process, and synapse organization, Figure S9A. Telomere shortening is associated with AD [77]. The substantia nigra is crucial for motor control and dopamine production, showing significant degeneration in AD [78]. The process of amyloid fibril formation involves cystatin‐B, microtubule‐associated protein tau, alpha‐synuclein, and peptidyl‐prolyl *cis*–*trans* isomerase FKBP1A proteins. Pathway analysis of those genes indicates high enrichment in primary genetic hyperlipidemia, hyperlipoproteinemia, progressive neurological disorders, and Parkinsonism, Figure S9B. Primary genetic hyperlipidemia and hyperlipoproteinemia have been linked to AD [79]. Microtubule‐associated protein tau, Parkinson disease protein 7 (PARK7), alpha‐synuclein, and beta‐synuclein are also proteins involved in Parkinson's disease pathways and neurodegeneration.

For proteoforms having higher abundance in healthy control as well as those exclusively identified in control brain samples (Table S3), the corresponding genes were highly enriched in processes essential for normal neuronal function, including chemical synaptic transmission, calcium ion‐related activities, long‐term synaptic potentiation, neurotransmitter transport, and axogenesis, Figure S9C. The axogenesis category included two truncated neurofilament light polypeptide proteoforms and a single proteoform of synaptosomal‐associated protein 25 (SNAP25) and microtubule‐associated protein 1A (MAP1A). Neurofilament light chain (NEFL) is a key structural component of the neuronal cytoskeleton and is recognized as a promising biomarker of AD and other neurodegenerative diseases because damaged nerve cells release NEFL into cerebrospinal fluid and blood [80]. Interestingly, pathway analysis revealed that highly expressed genes in control samples are highly enriched in motor dysfunction and progressive parkinsonism, Figure S9D, though these subjects did not display antemortem motor problems (see Section 2). Excitatory amino acid transporter 2 (EAAT2), alpha‐synuclein, beta‐synuclein, and microtubule‐associated protein tau are involved in motor dysfunction pathways. EAAT2 is a critical protein responsible for removing excess glutamate from synaptic spaces within the central nervous system. This transporter is predominantly found in glial cells, especially astrocytes, where it maintains proper glutamate homeostasis. Impaired EAAT2 function has been associated with multiple neurological conditions and neurodegenerative diseases [81].

The GO and pathway analysis revealed alterations in biological processes and pathways in AD compared to the control, providing insights into proteome‐wide dynamics in AD. Although the coverage of TDP is inherently limited compared to bottom‐up approaches, it uniquely captures intact proteoforms, including PTMs and proteolytic fragments, which may reflect disease‐specific processing events. For example, prior peptidomics studies have identified dysregulated proteolytic pathways in AD, such as elevated amyloid‐β fragments [82], tau‐derived peptides [83], and neuropeptide degradation [84]. Our top‐down data complements these findings by resolving intact proteoforms with combinations of PTMs and/or truncations that are often obscured in bottom‐up workflows.

### 3.4 Comparisons to Other TDP Studies

Fulcher et al. analyzed 103 frontal cortex samples of AD patients by RPLC‐FAIMS‐MS and identified 11,782 proteoforms, with 154 showing significant associations with clinicopathological phenotypes of AD [28]. The main goal of the study is to establish the connections between brain proteoforms and different pathological phenotypes, for example, amyloid plaque and cerebral amyloid angiopathy. In our pilot study here, we measured 10 human brain samples (5 ADs and 5 controls) using the SEC‐CZE‐MS/MS approach, aiming to determine proteoform profile differences between ADs and healthy controls and identify potential proteoform biomarkers of AD. We determined that Aβ42 and Aβ40 were the most abundant Aβ proteoforms in the AD samples, which agreed well with the literature [28].

Fulcher et al. identified about 2000 unique proteoforms from AD brain samples using RPLC‐FAIMS‐MS/MS [29]. The study focused on the evaluation and optimization of RPLC‐FAIMS‐MS/MS for the analysis of human brain proteoforms. In our study, we focused on discovering differentially expressed proteoforms between AD and healthy human brain samples using SEC‐CZE‐MS/MS, with the identification of over 3000 proteoforms and the determination of hundreds of differentially quantified proteoforms between AD and healthy human brain samples.

Our TDP approach enabled direct identification of proteolytic tau fragments that are central to AD pathogenesis, as extensively reviewed by Quinn et al. [30]. We identified eight phosphorylated and proteolytic tau (2N4R) proteoforms, with five being condition‐specific (four unique to AD, one unique to control). The three common tau proteoforms have much higher abundance in AD compared to control cases. The drastically different tau proteoform profiles between AD patients and healthy controls validate the pathological relevance of tau proteolysis in AD [30].

## 4 Conclusions

This pilot study represents the first quantitative TDP study of human AD and control brain samples using CZE‐MS/MS. The SEC‐CZE‐MS/MS platform successfully identified over 3000 unique proteoforms, revealing a profound proteoform remodeling in AD compared to control. We revealed bidirectional proteoform regulation between AD and control for multiple key genes in AD development, indicating the importance of proteoform‐specific quantitative measurement of human brains for a better understanding of the molecular mechanisms of AD. The altered biological processes and pathways are highly relevant to AD development and provide a deep view of the human brain proteoform changes between AD and control subjects. One main limitation of this study is the small number of human brain samples analyzed. In the near future, we will expand this study to include more human brain samples from control, early AD, and potentially late AD subjects.

## Conflicts of Interest

The authors declare no conflicts of interest.

## Supporting information




**Supporting File 1**: pmic70041‐sup‐0001‐SuppMat.docx

**Supporting File 2**: pmic70041‐sup‐0002‐SuppMat.xlsx

## Data Availability

The MS raw data have been deposited to the ProteomeXchange Consortium via the PRIDE partner [85] repository with the data set identifier PXD065459.
