# Supplementary material for: Coupling CZE, Liquid‐Phase Ion Mobility, to MS/MS for Quantitative Top‐Down Proteomics: Revealing Significant Proteoform Differences Between Healthy and Alzheimer's Disease Brains
Source: Proteomics. 2025 Sep 14;26(4):23–35. doi: 10.1002/pmic.70041 (PMC13048455; doi:10.1002/pmic.70041)
Supplement: Supplementary file 1 — Supporting File 1: pmic70041‐sup‐0001‐SuppMat.docx [file PMIC-26--s001.docx]

**Coupling CZE, liquid-phase ion mobility, to MS/MS for quantitative top-down proteomics: revealing significant proteoform differences between healthy and Alzheimer's disease brains**

Mehrdad Falamarzi Askarani,^1^ Fei Fang^1^, Scott E. Counts^2,^ *, Liangliang Sun^1,^ *

^1^Department of Chemistry, Michigan State University, 578 S Shaw Lane, East Lansing, Michigan 48824, USA

^2^Department of Translational Neuroscience, College of Human Medicine, Michigan State University, 400 Monroe Avenue NW, Grand Rapids, MI 49503, USA

* Corresponding Authors.

Scott E. Counts, Email: [countssc@msu.edu](mailto:countssc@msu.edu); Phone :  616-234-0997

Liangliang Sun, Email: [lsun@chemistry.msu.edu](mailto:lsun@chemistry.msu.edu); Phone: 517-353-0498


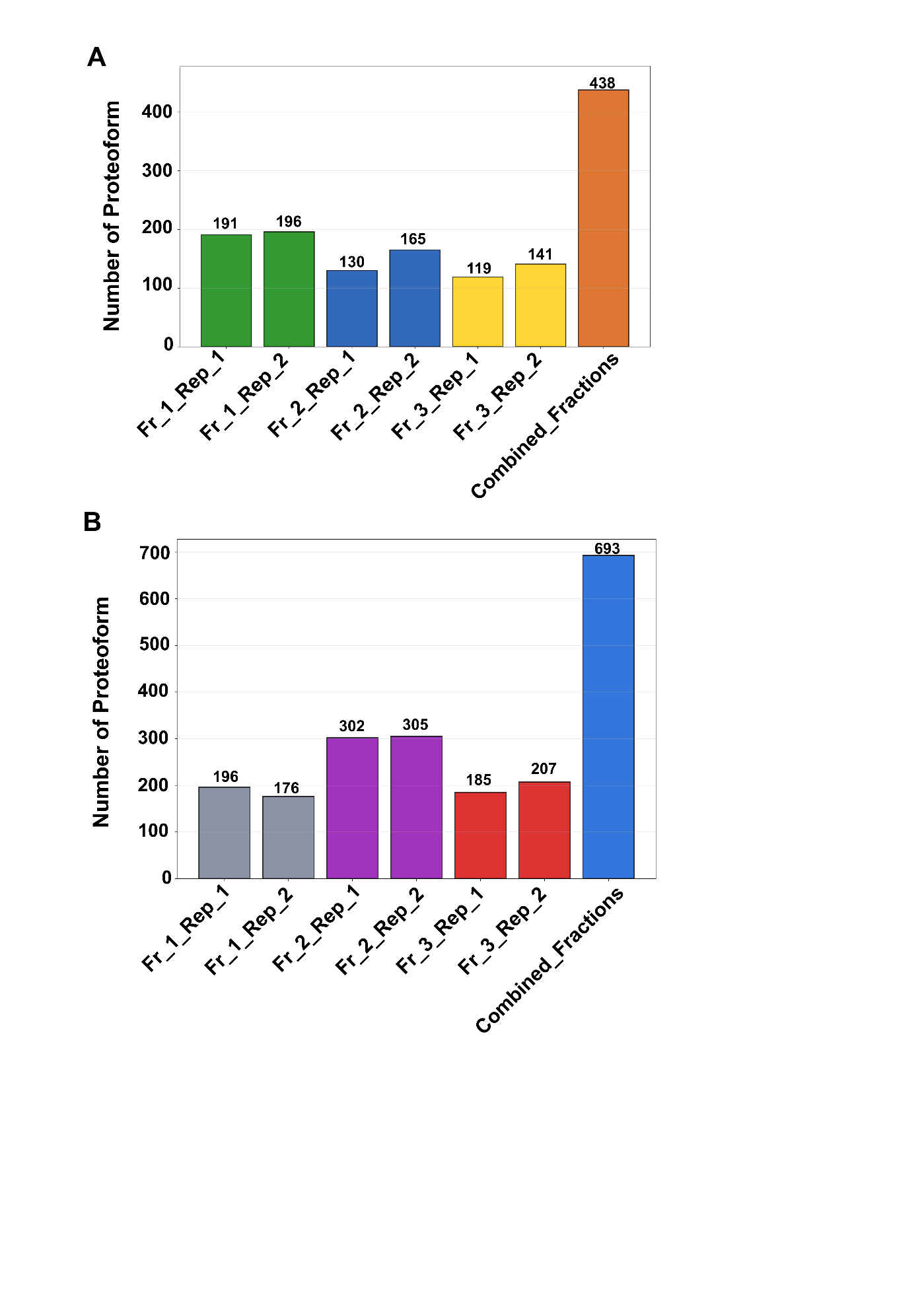


**Figure S1.** Proteoform distribution in SEC fractions from representative AD and control samples.
(A) Bar plot showing proteoform counts across SEC fractions for an AD sample (AD2). (B) Bar plot showing proteoform counts across SEC fractions for a healthy control sample (H1). For both samples, individual SEC fractions (F1-F3) and combined totals are shown, demonstrating improved proteome coverage through fractionation (n = 2 technical replicates per fraction).


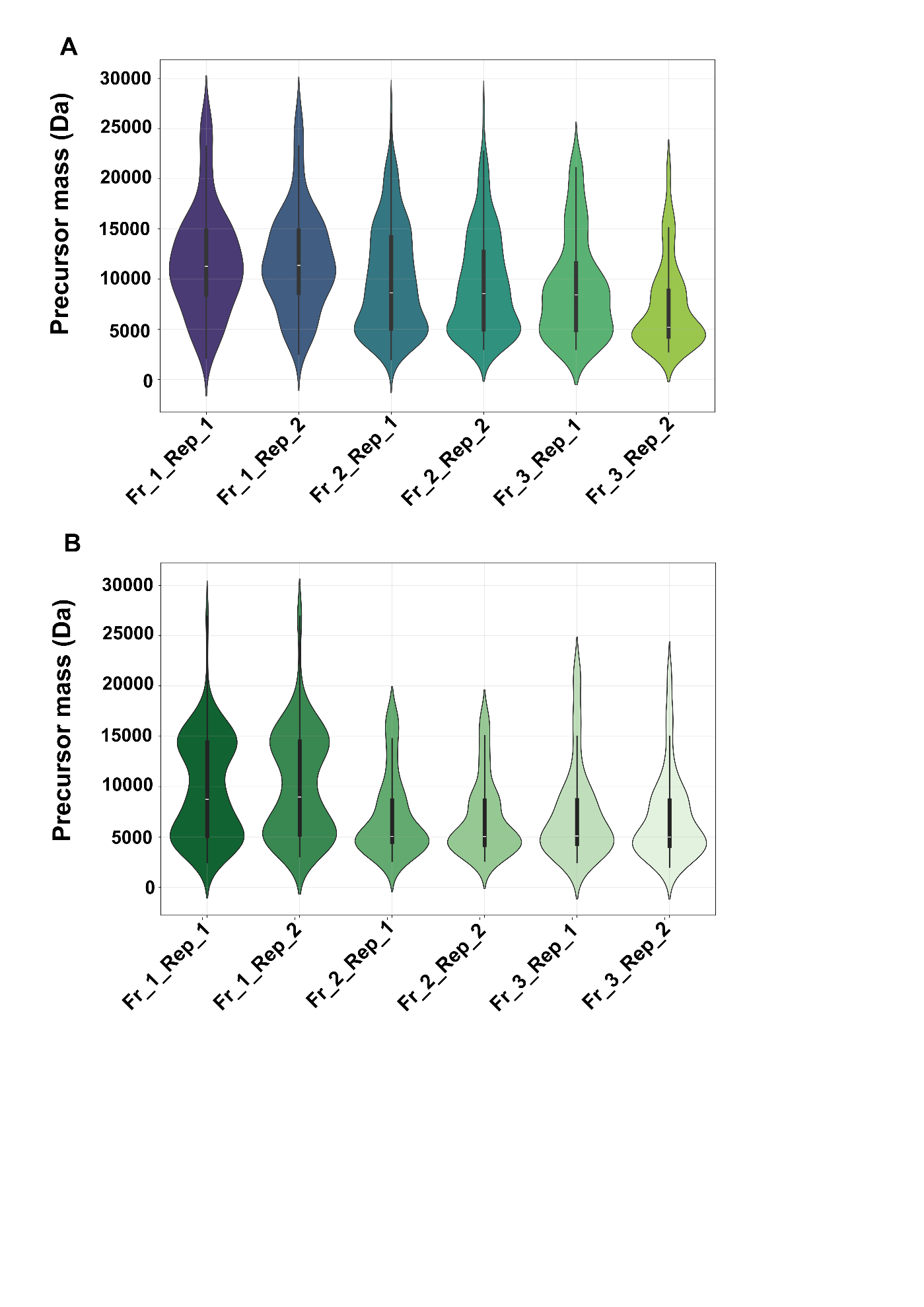


**Figure S2.** Mass distribution of proteoforms across SEC fractions analyzed by CZE-MS/MS. Violin plots showing precursor mass distributions for proteoforms identified in three SEC fractions from (A) AD sample AD2 and (B) healthy control H1. Technical replicates demonstrate progressive mass decrease from fraction 1 to 3, confirming effective size-based separation by SEC.


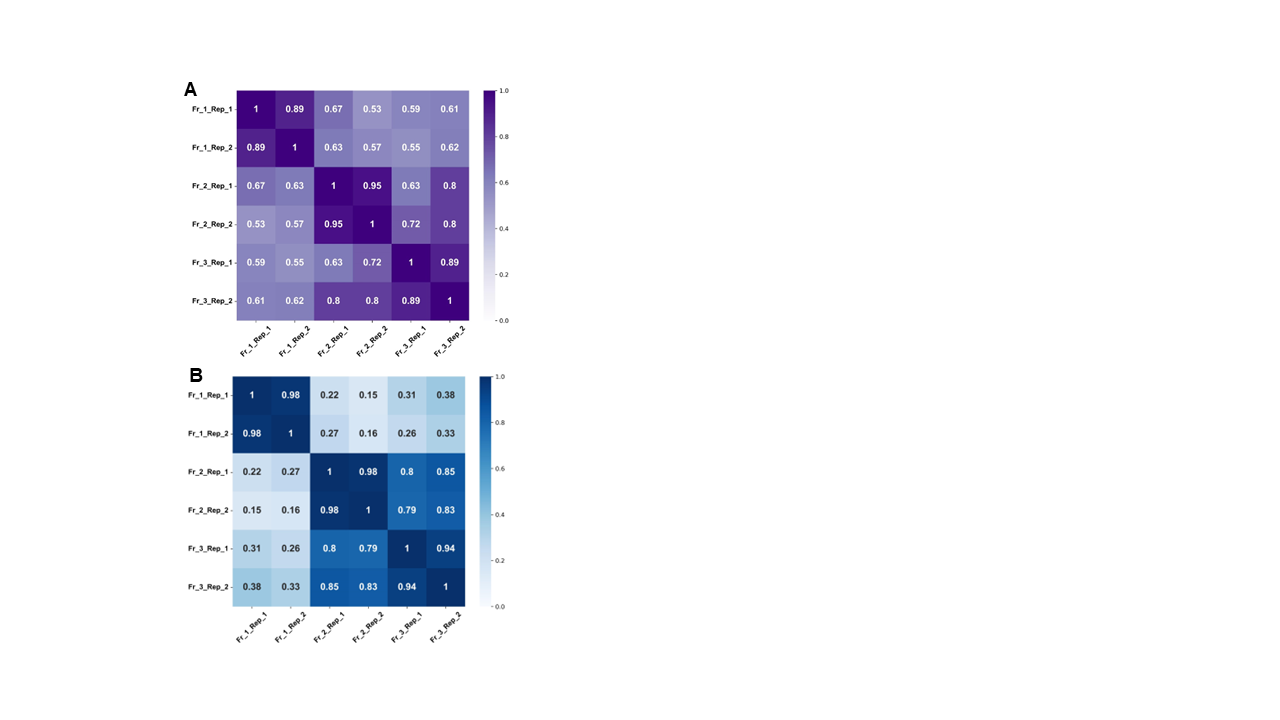


**Figure S3.** Linear correlation analysis of proteoform intensity across SEC fractions in representative AD and healthy control samples. (A) Heatmap displaying pairwise Pearson correlation coefficients of SEC fractions (Fr1-Fr3) and technical replicates (Rep1-Rep2) for an AD sample (AD2). (B) Heatmap displaying pairwise Pearson correlation coefficients of SEC fractions (Fr1-Fr3) and technical replicates (Rep1-Rep2) for all SEC fractions and technical replicates for a healthy control sample (H1). Strong linear correlations between technical replicates of the same SEC fraction indicate high reproducibility of CZE-MS/MS, while weak inter-fraction correlations demonstrate the effective separation of SEC for proteoforms. The color gradient represents the scale of the Pearson correlation coefficient.


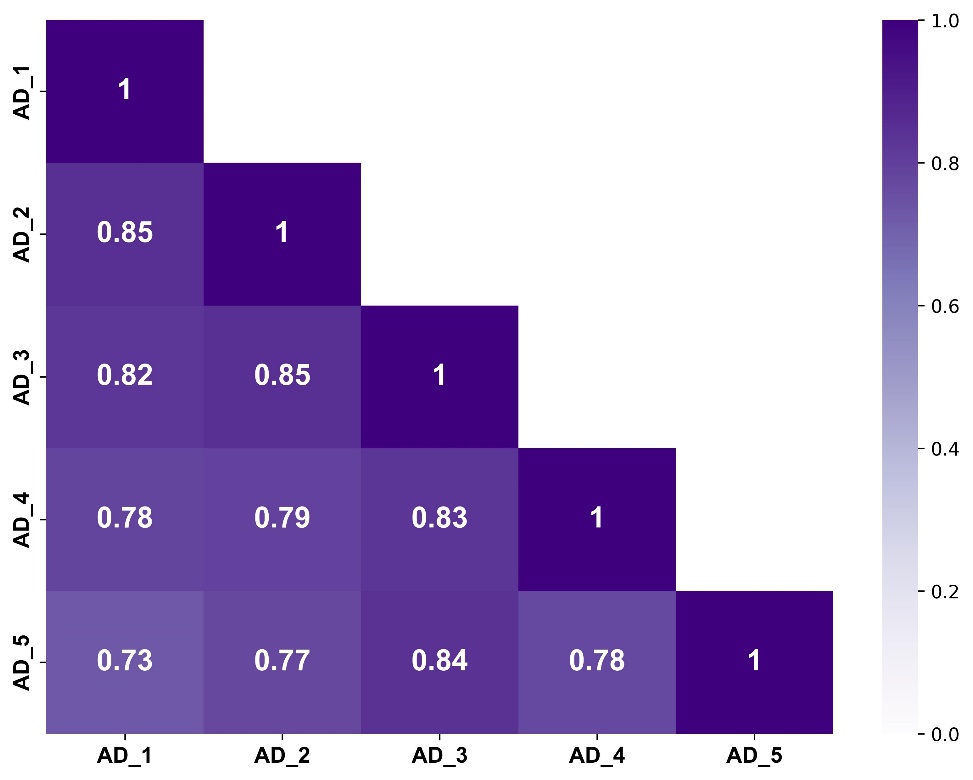

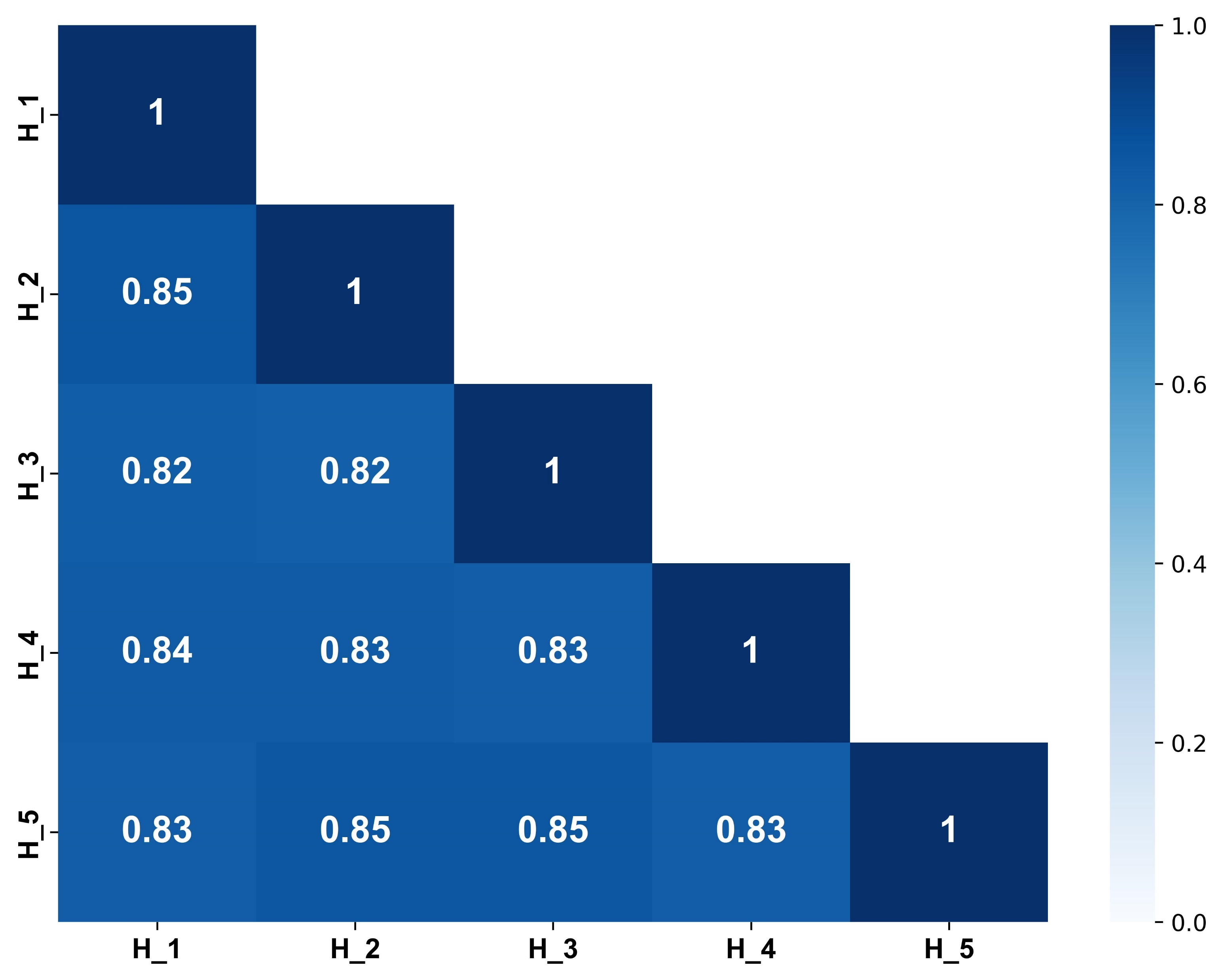


**A**

**B**

**Figure S4.** Pairwise Pearson correlation of proteoform intensity across biological replicates of AD (A) and healthy control (B) samples. AD_1 to AD_5 are the five biological replicates of AD, and H_1 to H_5 are the five biological replicates of the healthy control. The values in the heat maps represent the pairwise Pearson correlation coefficients, with a higher value indicating a stronger linear correlation.


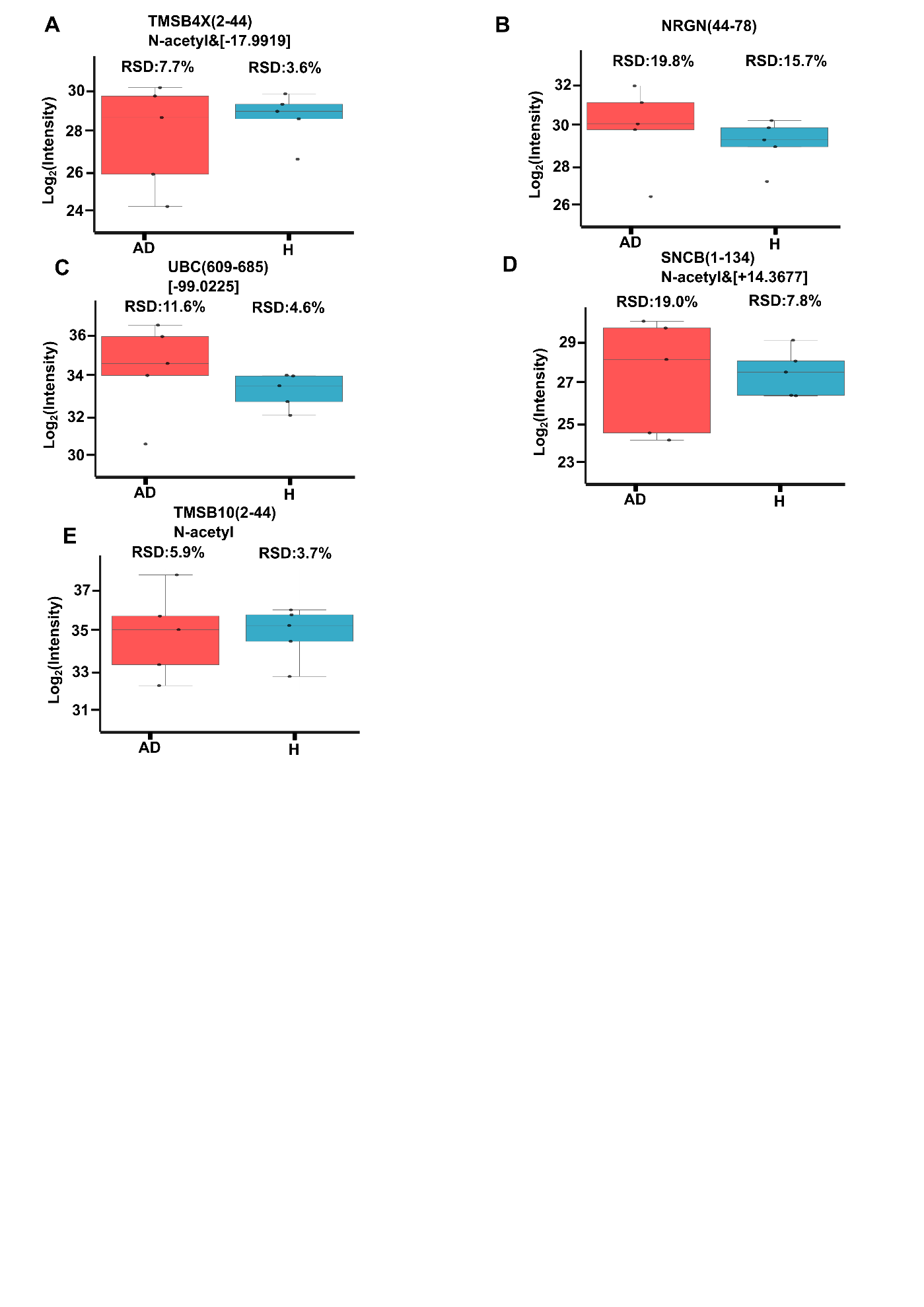


**Figure S5**. Box-plots of intensities of five example proteoforms showing substantially higher intensity variations in AD samples (n=5) compared to healthy controls (H, n=5). The gene names, the starting and ending amino acid numbers of the proteoforms, mass shifts or post-translational modifications (PTMs), and relative standard deviations (RSDs) of proteoform intensity are labelled on the figures. For example, the *TMSB4X* proteoform covering 2-44 amino acids (A) has an N-terminal acetylation PTM, and the RSDs of proteoform intensity are 7.7% and 3.6% for AD samples and healthy controls, respectively. As another example, the *UBC* proteoform carrying one -99.0225 Da mass shift (C) has an 11.6% and 4.6% RSD of proteoform intensity for AD and healthy control samples.


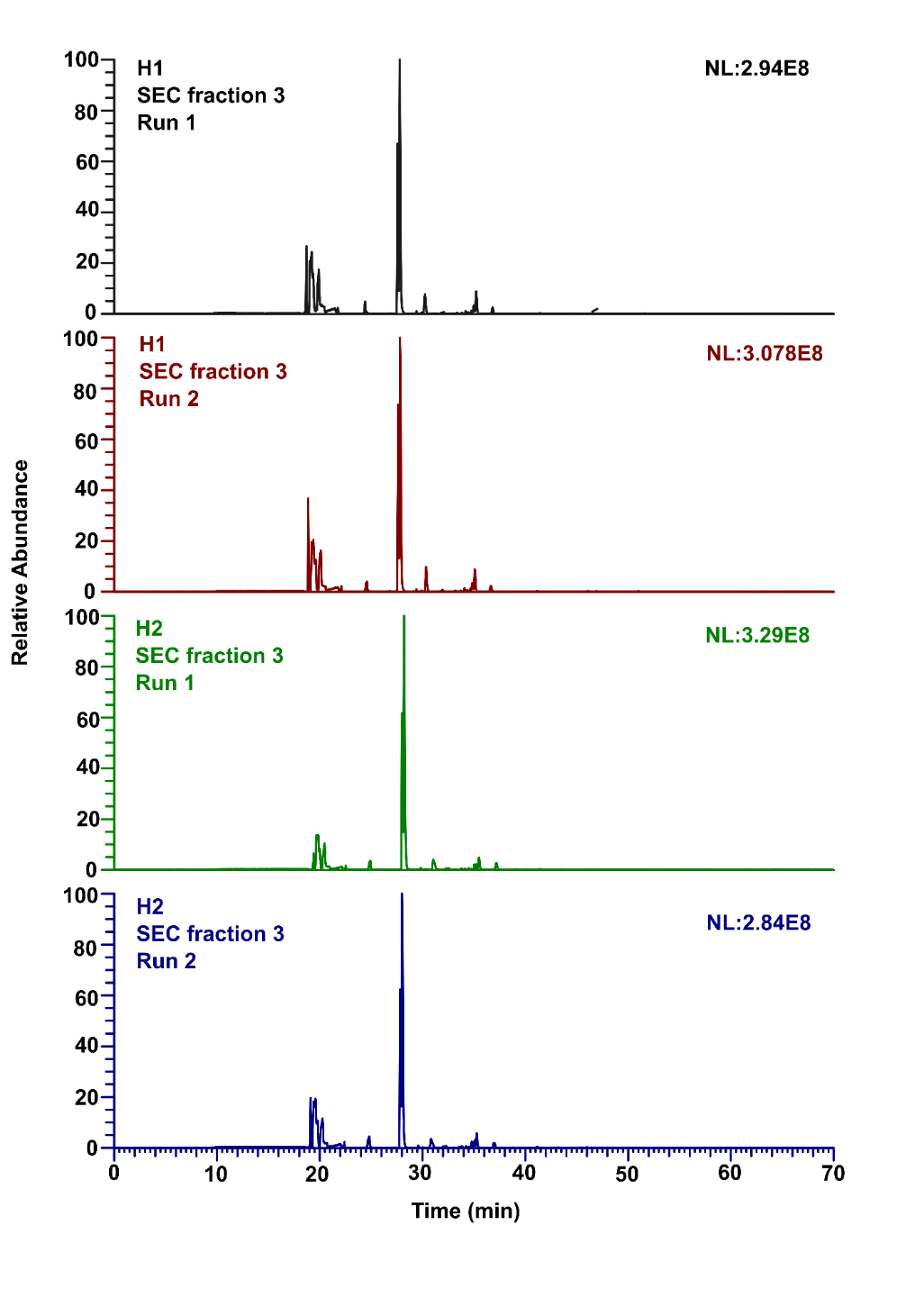


**Figure S6.** Base peak electropherograms of two healthy control samples (H1 and H2). The duplicate CZE-MS/MS runs of one SEC fraction (fraction 3) are shown. The highly consistent profiles between duplicate runs demonstrate the excellent reproducibility of our CZE-MS/MS analytical platform.


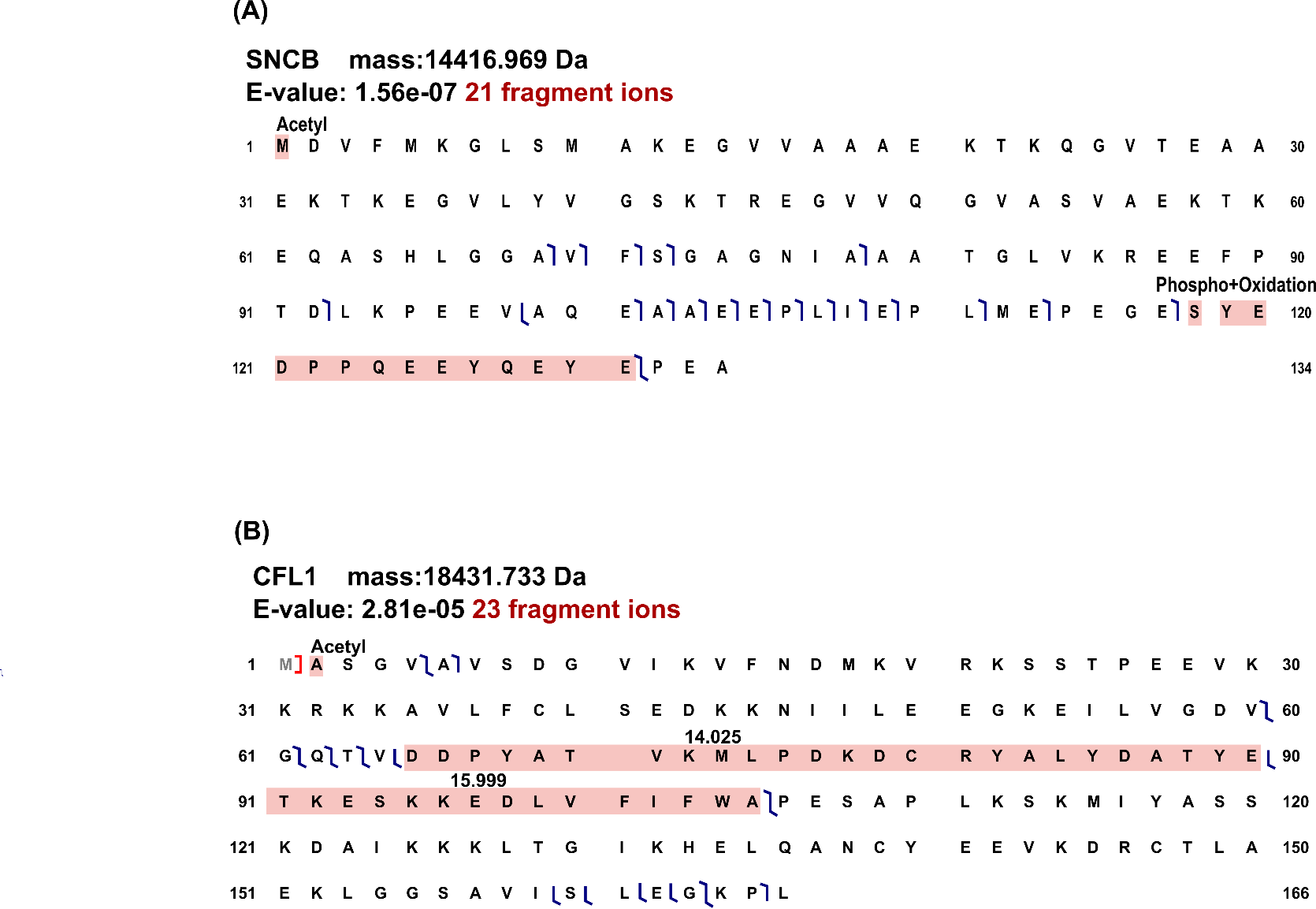


**Figure S7.** Amino acid sequences and MS/MS fragmentation patterns of two identified proteoforms. A) SNCB proteoform with N-terminal acetylation, oxidation, and phosphorylation modifications. B) CFL1 proteoform featuring N-terminal acetylation, and mass shifts of 14.025 Da and 15.999 Da, likely corresponding to methylation and oxidation modifications, respectively. The marked amino acid residue regions indicate the potential modification sites. The exact modification sites cannot be determined in most cases due to the limited backbone cleavage coverage.


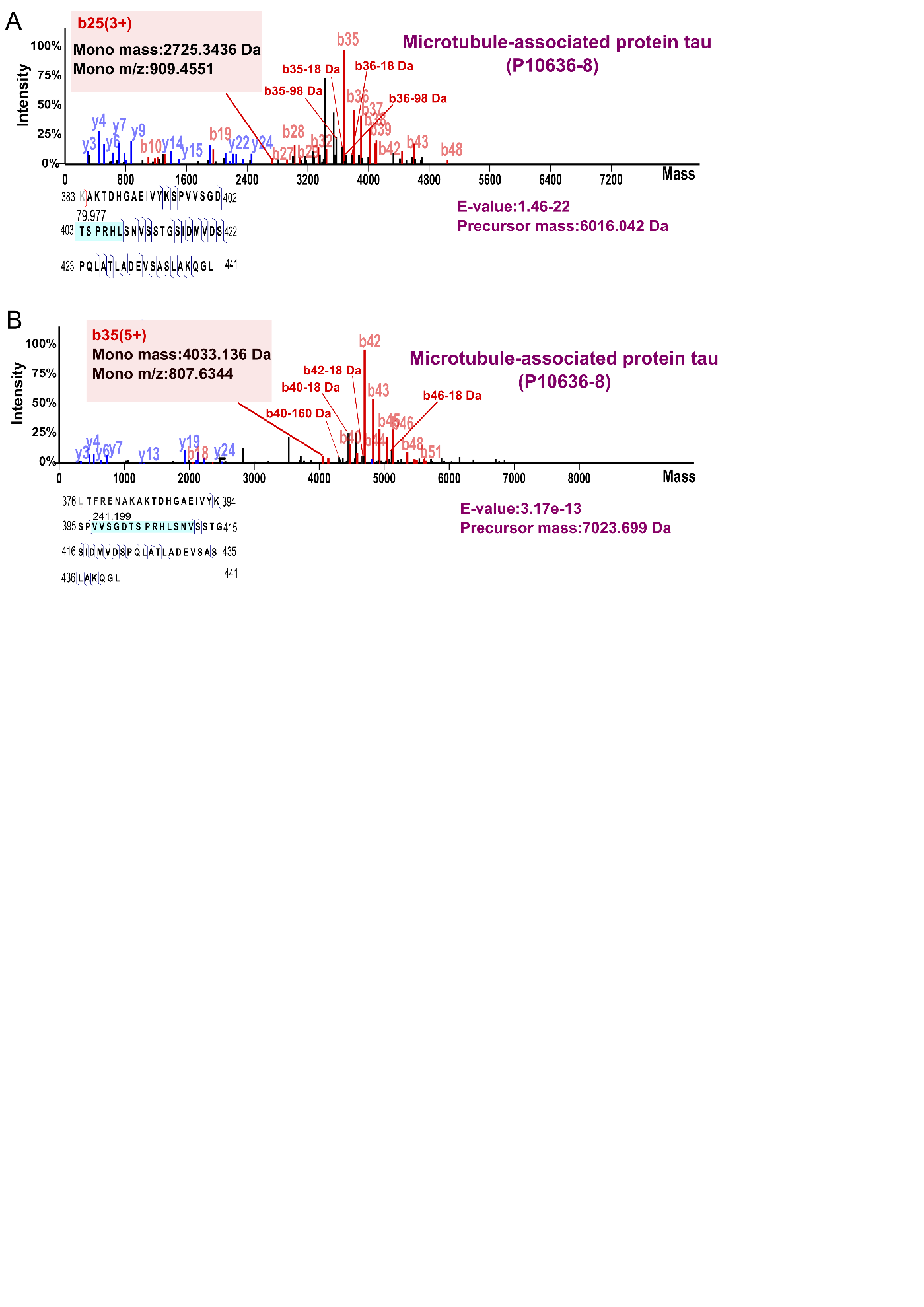


**Figure S8**. The annotated MS/MS spectra and fragmentation patterns of two Microtubule-associated protein tau (2N4R) proteoforms with one phosphorylation site (A) or with three phosphorylation sites(B). Red boxes highlight the sequence regions containing specific post-translational modification sites. The fragment ions with 98-Da,160-Da, and 18-Da neutral losses further confirm the phosphorylation modification on the two 2N4R proteoforms.

**
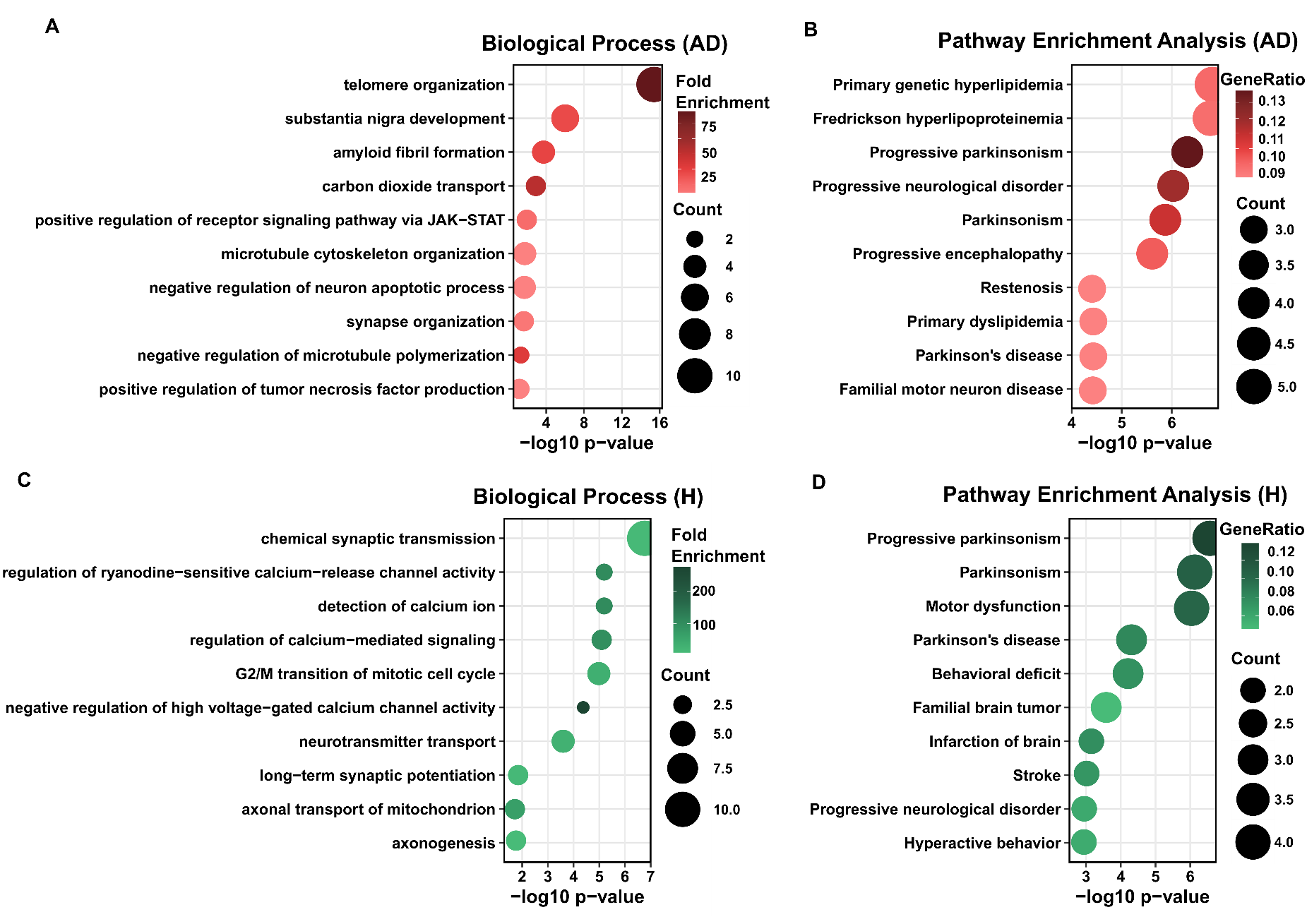
**

**Figure S9.** Gene ontology and pathway enrichment analysis of differentially quantified proteoforms, as well as those exclusively identified in AD or healthy brain samples. Enriched Biological Processes (A) and pathways (B) of genes corresponding to up-regulated proteoforms in AD and proteoforms that were exclusively identified in AD. Enriched Biological Processes (C) and pathways (D) of genes corresponding to up-regulated proteoforms in healthy control samples and proteoforms that were exclusively identified in control samples. The X-axis represents the -log₁₀(*p*-value), indicating the significance of enrichment, while the Y-axis lists the specific Biological Processes and pathway enrichment analysis. Bubble size corresponds to the number of genes involved, and the color gradient reflects the fold enrichment, with higher values indicating stronger enrichment.

| **Table S1.** Subject demographics and clinical pathologic variables. | | | | |  |
| --- | --- | --- | --- | --- | --- |
|  | Control | AD | (*p* value)* |  |  |
|  | (n=5) | (n=5) |  |  |  |
| Age at death (years) |  |  |  |  |  |
| Mean ± SD | 82.2 ± 7.7 | 82.4 ± 5.9 | 0.96 |  |  |
| (Range) | (73-91) | (75-89) |  |  |  |
| No. (%) Males | 3 (60%) | 2 (40%) | 0.58^‡^ |  |  |
| Education  (years) |  |  |  |  |  |
| Mean ± SD | 15.25± 2.2 | 15.92± 2.4 | 0.88 |  |  |
| (Range) | (13-18) | (12-20) |  |  |  |
| Postmortem Interval (hours) |  |  |  |  |  |
| Mean ± SD | 9.2 ± 2.9 | 9.4 ± 2.5 | 0.91 |  |  |
| (Range) | (7-14) | (7-12) |  |  |  |
| MMSE | |  |  |  |  |
| Mean ± SD | 29 ± 1 | 21.6 ± 1.8 | <0.0001 |  |  |
| (Range) | (28-30) | (19-24) |  |  |  |
| Braak Stage |  |  |  |  |  |
| I-II | 5 | 0 | 0.0004 |  |  |
| III-IV | 0 | 5 |  |  |  |
| V-VI | 0 | 0 |  |  |  |

*Student’s t test (all comparisions but % male); Shapiro -Wilk test used for normality

^‡^Chi-square test

Abbreviations: MMSE, Mini-Mental State Exam; SD, standard deviation

# **Table S2.** Proteoforms exclusively detected in AD samples via SEC-CZE-MS/MS analysis.

| Protein accession | Proteoform | Precursor mass | First residue | Last residue | Gene | E-value |
| --- | --- | --- | --- | --- | --- | --- |
| sp\|P62937\|PPIA_HUMAN | M.VNPTVFFDIAVDGEPLGRVSFELFADKVPKTAENFRALSTGEKGFGYKGSCFHRIIPGFMCQGGDFTRHNGTGGKSIYGEKFEDENFILKHTGPGILSMANAGPNTNGSQFFICTAKTEWLDGKHVVFGKVKEGMNIVEAMERFGSRNGKTSKKITIADCGQLE. | 17871.0 | 2 | 165 | PPIA | 2.93e-12 |
| sp\|P68871\|HBB_HUMAN | M.VHLTPEEKSAVTALWGKVNVDEVGGEALGRLLVVYPWTQRFF(E)[-20.8893]SFGDLSTPDAVMGNPKVKAHGKKVLGAFSDGLAHLDNLKGTFATLSELHCDKLHVDPENFRLLGNVLVCVLAHHFGKEFTPPVQAAYQKVVAGVANALAHKYH. | 15835.0 | 2 | 147 | HBB | 4.68e-40 |
| sp\|P69905\|HBA_HUMAN | .MVLSPADKTNVKAAWGKVGAHAGEYGAEALE(RMF)[-132.0163]LSFPTTKTYFPHFDLSHGSAQVKGHGKKVADALTNAVAHVDDMPNALSALSDLHAHKLRVDPVNFKLLSHCLLVTLAAHLPAEFTPAVHASLDKFLASVSTVLTSKYR. | 15115.0 | 1 | 142 | HBA1 | 1.66e-11 |
| sp\|P30086\|PEBP1_HUMAN | M.PVDLSKWSGPLSLQEVDEQPQHPLHVTYAGAAVDELGKVLTPTQVKNRPTSISWDGLDSGKLYTLVLTDPDAPSRKDPKYREWHHFLVVNMKGNDISSGT(V)[+4.0948]LSDYVGSGPPKGTGLHRYVWLVYEQDRPLKCDEPILSNRSGDHRGKFKVASFRKKYELRAPVAGTCYQAEWDDYVPKLYEQLSGK. | 20917.9 | 2 | 187 | PEBP1 | 1.4e-26 |
| sp\|P14174\|MIF_HUMAN | M.PMFIVNTNVPRASVPDGFLSELTQQLAQATGKPPQYIAVHVVPDQLMAFGGSSEPCALCSLHSIGKIGGAQNRSYSKLLCGLLAERLRISPDRVYINYYDMNAANVGWNNSTF(A)[+30.0831]. | 12368.3 | 2 | 115 | MIF | 6.21e-22 |
| sp\|P68871\|HBB_HUMAN | M.VHLTPEEKSAVTALWGKVNVDEVGGEALGRLLV(VYPWTQRFFESFGDLST)[+29.5959]PDAVMGNPKVKAHGKKVLGAFSDGLAHLDNLKGTFATLSELHCDKLHVDPENFRLLGNVLVCVLAHHFGKEFTPPVQAAYQKVVAGVANALAHKYH. | 15885.5 | 2 | 147 | HBB | 4.68e-40 |
| sp\|P23528\|COF1_HUMAN | M.[Acetyl]-ASGVAVSDGVIKVFNDMKVRKSSTPEEVKKRKKAVLFCLSEDKKNIILEEGKEILVGDVGQTVDDPYATFVKMLPDKDCRYALYDATYETKESKKEDLVFIFWAPESAPLKSKMIYASSKDAIKKKLTGIKHELQANCYEEVKDRCTLAEKLGGSAVISLEGKPL. | 18400.7 | 2 | 166 | CFL1 | 1.17e-08 |
| sp\|P69905\|HBA_HUMAN | .M(VL)[-32.9585]SPADKTNVKAAWGKVGAHAGEYGAEALERMFLSFPTTKTYFPHFDLSHGSAQVKGHGKKVADALTNAVAHVDDMPNALSALSDLHAHKLRVDPVNFKLLSHCLLVTLAAHLPAEFTPAVHASLDKFLASVSTVLTSKYR. | 15214.0 | 1 | 142 | HBA1 | 1.66e-11 |
| sp\|P07737\|PROF1_HUMAN | M.[Acetyl]-AGWNAYIDNLMADGTCQDAAIVGYKDSPSVWAAVPGKTFVNITPAEVGVLVGKDRSSFYVNGLTLGGQKCSVIRDSLLQDGEFSMDLRTKSTGGAPT(FNVTVT)[+1.1064]KTDKTLVLLMGKEGVHGGLINKKCYEMASHLRRSQY. | 14955.8 | 2 | 140 | PFN1 | 2.08e-19 |
| sp\|Q16143\|SYUB_HUMAN | .[Acetyl]-MDVFMKGLSMAKEGVVAAAEKTKQGVTEAAEKTKEGVLYVGSKTREGVVQGVASVAEKTKEQASHLGGAVFSGAGNIAAATGLVKREEFPTDLKPEEVAQEAAEEPLIE(PLMEPEGESYED)[-3.5544]PPQEEYQEYEPEA. | 14316.2 | 1 | 134 | SNCB | 4.89e-26 |
| sp\|P10599\|THIO_HUMAN | .MV(KQIESKTAFQEALDAAGDKLVVVDFSATWCGPCKMIKPFFHSLSEKYSNV)[-134.0115]IFLEVDVDDCQDVASECEVKCMPTFQFFKKGQKVGEFSGANKEKLEATINELV. | 11596.8 | 1 | 105 | TXN | 1.05e-05 |
| sp\|Q99497\|PARK7_HUMAN | M.[Acetyl]-ASKRALVILAKGAEEMETVIPVDVMRRAGIKVTVAGLAGKDPVQCSRDVVICPDASLEDAKKEGPYDVVVLPGGN(LGAQNLSESAAVKEILKEQENRKGLIAAICA)[+191.5760]GPTALLAHEIGFGSKVTTHPLAKDKMMNGGHYTYSENRVEKDGLILTSRGPGTSFEFALAIVEALNGKEVAAQVKAPLVLKD. | 19979.6 | 2 | 189 | PARK7 | 7.500000000000001e-32 |
| sp\|P60983\|GMFB_HUMAN | M.[Acetyl]-SESLVVCDVAEDLVEKLRKFRFRKETNNAAIIMKIDKDKRLVVLDEELEGISPDELKDELPERQPRFIVYSYKYQHDDGRVSYPLCFIFSSPVGCKPEQQMMYAGSKNKLVQTAELTKVFEIRNTEDLTEEWLREKLGFFH. | 16613.6 | 2 | 142 | GMFB | 6.19e-09 |
| sp\|P68871\|HBB_HUMAN | M.VHLTPEEKSAVTALWGKVNVDEVGGEALGRLLV(V)[+10.7374]YPWTQRFFESFGDLSTPDAVMGNPKVKAHGKKVLGAFSDGLAHLDNLKGTFATLSELHCDKLHVDPENFRLLGNVLVCVLAHHFGKEFTPPVQAAYQKVVAGVANALAHKYH. | 15867.0 | 2 | 147 | HBB | 4.68e-40 |
| sp\|P62937\|PPIA_HUMAN | M.VNPTVFFDIAVDGEPLGRVSFELFADKVPKTAENFRALSTGEKGFGYKGSCFHRI(IPGFMCQGGDFTRHNGTGGKSIYGEKFEDENFILKHTGPGIL)[Methyl;Oxidation]SMANAGPNTNGSQFFICTAKTEWLDGKHVVFGKVKEGMNIVEAMERFGSRNGKTSKKITIADCGQLE. | 17900.0 | 2 | 165 | PPIA | 2.93e-12 |
| sp\|Q9UI15\|TAGL3_HUMAN | M.[Acetyl]-ANRGPSYGLSREVQEKIEQKYDADLENKLVDWIILQCAEDIEHPPPGRAHFQKWLMDGTVLCKLINSLYPPGQEPIPKISESKMAFKQMEQISQFLKAAETYGVRTTDIFQTVDLWEGKDMAAVQRTLMALGSVAVTKDDGCYRGEPSWFHRKAQQNRRGFSEEQLRQGQNVIGLQMGSNKGASQAGMTGYGMPRQ.I | 22125.2 | 2 | 197 | TAGLN3 | 2.64e-08 |
| sp\|P68871\|HBB_HUMAN | M.VHLTPEEKSAVTALWGKVNVDEVGGEALGRLLVVYPWTQRFFESFGD(L)[+7.6438]STPDAVMGNPKVKAHGKKVLGAFSDGLAHLDNLKGTFATLSELHCDKLHVDPENFRLLGNVLVCVLAHHFGKEFTPPVQAAYQKVVAGVANALAHKYH. | 15865.0 | 2 | 147 | HBB | 4.68e-40 |
| sp\|P30086\|PEBP1_HUMAN | M.PVDLSKWSGPLSLQEVDEQPQHPLHVTYAGAAVDELGKVLTPTQVKNRPTSISWDGLDSGKLYTLVLTDPDAPSRKDPKYREWHHFLVVNMKGNDISSGT(V)[+97.1025]LSDYVGSGPPKGTGLHRYVWLVYEQDRPLKCDEPILSNRSGDHRGKFKVASFRKKYELRAPVAGTCYQAEWDDYVPKLYEQLSGK. | 21010.7 | 2 | 187 | PEBP1 | 1.4e-26 |
| sp\|P00441\|SODC_HUMAN | M.[Acetyl]-ATKAVCVLKGDGPVQGIINFEQKESNGPVKVWGSIKGLTEGLHGFHVHEFGDNTAGCTSAGPHFNPLSRKHGGPKDEERHVGDLGNVTADKDGVADVSIEDSVISLSGDHCIIGRTLVVHEKADDLGKGGNEESTKTGNAGSRLACGVIG(IAQ)[-16.9711]. | 15819.0 | 2 | 154 | SOD1 | 1.22e-17 |
| sp\|P60174\|TPIS_HUMAN | M.APSRKFFVGGNWKMNGRKQSLGELIGTLNAAKVPADTEVVCAPPTAYIDFARQKLDPKIAVAAQNCYKVTNGAFT(GEISPGM)[-284.5294]IKDCGATWVVLGHSERRHVFGESDELIGQKVAHALAEGLGVIACIG.E | 13261.5 | 2 | 129 | TPI1 | 0.001369147 |
| sp\|O75347\|TBCA_HUMAN | M.[Acetyl]-ADPRVRQIKIKTGVVKRLVKEKVMYEKEAKQQEEKIEKMRAEDGENYDIKKQAEILQESRMMIPDCQRRLEAAYLDLQRILENEKDLEEAEEYKEARLVLDSVKLEA. | 12757.7 | 2 | 108 | TBCA | 2.28e-15 |
| sp\|P0CG47\|UBB_HUMAN | .MQIFVKTLTGKTITLEVE(PSD)[+99.0611]TIENVKAKIQDKEGIPPDQQRLIFAGKQLEDGRTLSDYNIQKESTLHLVLRL.R | 8387.48 | 1 | 73 | UBB | 1.8e-20 |
| sp\|P09382\|LEG1_HUMAN | M.[Acetyl]-ACGLVASNLNLKPGECLRVRGEVAPD(AKSFVLNLGKDSNNLCLHFNPRFNAHGDAN)[-0.9501]TIVCNSKDGGAWGTEQREAVFPFQPGSVAEVCITFDQANLTVKLPDGYEFKFPNRLNLEAINYMAADGDFKIKCVAFD. | 14615.4 | 2 | 135 | LGALS1 | 3.3e-14 |
| sp\|Q15121\|PEA15_HUMAN | .(MA)[-92.8185]EYGTLLQDLTNNITLEDLEQLKSACKEDIPSEKSEEITTGSAWFSFLESHNKLDKDNLSYIEHIFEISRRPDLLTMVVDYRTRVLKISEEDELDTKLTRIPSAKKYKDIIRQPSEEEIIKLAPPPKKA. | 14939.0 | 1 | 130 | PEA15 | 0.00106461 |
| sp\|P30044\|PRDX5_HUMAN | A.PIKVGDAIPAVEVFEGEPGNKVNLAELFKGKKGVLFGVPGAFTPGCSKTHLPGFVEQAEALKAKGVQVVACLSVNDAFVTGEWGRAHKAEGKVRLLADPTGAFGKETDLLLDDSLVSIFGNRRLKRFSMVVQDGIVKALNVEPDGTGLTCSLAPNIISQL. | 16818.0 | 55 | 214 | PRDX5 | 8.17e-18 |
| sp\|P06703\|S10A6_HUMAN | M.[Acetyl]-ACPLDQA(IGLLVAIFHKYSGREGDKHTLSKKELKELIQKELTIGSKLQDAEIARLMEDLDRNKDQEVNFQEYVTFL)[+97.2561]GALALIYNEALKG. | 10182.4 | 2 | 90 | S100A6 | 3.43e-15 |
| sp\|P62328\|TYB4_HUMAN | M.[Acetyl]-SDKPDMAEIEKFDKSKLKKTETQEKNPLPSKETIEQEKQAGE(S)[+97.0568]. | 5058.47 | 2 | 44 | TMSB4X | 6.66e-33 |
| sp\|P30086\|PEBP1_HUMAN | M.(KGNDISSGTVL)[-261.3271]SDYVGSGPPKGTGLHRYVWLVYEQDRPLKCDEPILSNRSGDHRGKFKVASFRKKYELRAPVAGTCYQAEWDDYVPKLYEQLSGK. | 10456.9 | 93 | 187 | PEBP1 | 4.44e-07 |
| sp\|P30086\|PEBP1_HUMAN | M.PVDLSKWSGPLSLQEVDEQPQHPLHVT(YAGAAVDELGKVLTPTQVKNRPTSISWDGLDSGKLYTLVLTDPDAPSRKDPKYREWHHFLVVNMKGNDISSGTVL)[-0.2277]SDYVGSGPPKGTGLHRYVWLVYEQDRPLKCDEPILSNRSGDHRGKFKVASFRKKYELRAPVAGTCYQAEWDDYVPKLYEQLSGK. | 20911.0 | 2 | 187 | PEBP1 | 1.4e-26 |
| sp\|Q15370\|ELOB_HUMAN | .[Acetyl]-MDVFLMIRRHKTTIFTD(AKESSTVFELKRIVEGILKRPPDEQRLYKDDQ)[-0.9021]LLDDGKTLGECGFTSQTARPQAPATVGLAFRADDTFEALCIEPFSSPPELPDVMKPQDSGSSANEQAVQ. | 13166.8 | 1 | 118 | ELOB | 1.16e-08 |
| sp\|P06703\|S10A6_HUMAN | M.[Acetyl]-ACPLDQAI(GLL)[+31.0479]VAIFHKYSGREGDKHTLSKKELKELIQKELTIGSKLQDAEIARLMEDLDRNKDQEVNFQEYVTFLGALALIYNEALKG. | 10114.4 | 2 | 90 | S100A6 | 3.43e-15 |
| sp\|P0CG47\|UBB_HUMAN | .MQIFVKTLTGKTITL(E)[+194.1344]VEPSDTIENVKAKIQDKEGIPPDQQRLIFAGKQLEDGRTLSDYNIQKESTLHLVLRLRGG.M | 8754.58 | 1 | 76 | UBB | 4.48e-31 |
| sp\|P63167\|DYL1_HUMAN | .[Acetyl]-MCD(RKAVIKNADMSEEMQQDSVECATQALEKYNIEKDIAAHIKKEFDKKYNPTWHCIVGRNFGSYVTHETKHFIYFYL)[-48.0968]GQVAILLFKSG. | 10352.0 | 1 | 89 | DYNLL1 | 6.64e-10 |
| sp\|P60174\|TPIS_HUMAN | M.APSRKFFVGGNWKMNGRKQSLGELIGTLNAAKVPADTEVVC(APPTAYIDFARQKLDPKIAVAAQNCYKVTNG)[-124.9745]AFTGEISPGMIK.D | 8840.66 | 2 | 85 | TPI1 | 8.58e-07 |
| sp\|P0CG48\|UBC_HUMAN | G.MQIFVKTLTGKTITLEVEPSDTIENVKAKIQDKEGIPPD(QQRLIFAGKQLEDGRTLSDY)[-230.0611]NIQKESTLHLVLRLRGGV. | 8429.64 | 609 | 685 | UBC | 5.7299999999999996e-24 |
| sp\|Q9P1F3\|ABRAL_HUMAN | .MNVDHEVNLLVE(EIHRLGSKNADGKLSVKFGVLFRDDKCANLFEALVGTLKAAKRRKIVTYPG)[+41.0325]ELLLQGVHDDVDIILLQD. | 9092.97 | 1 | 81 | ABRACL | 8.76e-10 |
| sp\|P07737\|PROF1_HUMAN | M.[Acetyl]-AGWNAYIDNL(MADGTCQDAAIVGYKDSPSVWAAV)[+31.2071]PGKTFVNITPAEVGVLVGKDRSSFYVNGLTLGGQKCSVIRDSLLQDGEFSMDLRTKSTGGAPTFNVTVTKTDKTLVLLMGKEGVHGGLINKKCYEMASHLRRSQY. | 14985.8 | 2 | 140 | PFN1 | 2.08e-19 |
| sp\|P14174\|MIF_HUMAN | M.PMFIVNTNVPRASVPDGFLSELTQQLAQATGKPPQYIAVHVVPDQLMAFGGSSEPCALCSLHSIG(KIGGAQNRSYSKLLCGLLAERLRISPDRVYINYYDMNA)[+15.0682]ANVGWNNSTFA. | 12352.4 | 2 | 115 | MIF | 6.21e-22 |
| sp\|P30046\|DOPD_HUMAN | M.PFLELDTNLPANRVPAGLEKRLCAAAASILGKPADRVNVTVRPGLAMALSGSTEPCAQLSISSIGVVGTAEDNRSHSAHFFEFLTKELALGQDRILIRFFPLESWQIGKIGTVMT.F | 12312.6 | 2 | 116 | DDT | 5.23e-11 |
| sp\|P0CG47\|UBB_HUMAN | .MQIFVKTLTGKTITLEVEP(SDTIENVKAKIQDKEGIPPD)[+97.0699]QQRLIFAGKQLEDGRTLSDYNIQKESTLHLVLR.L | 8274.4 | 1 | 72 | UBB | 1.75e-23 |
| sp\|P31949\|S10AB_HUMAN | M.[Acetyl]-AKISSPTETERCIESLIAVFQKYAGKDGYNYTLSKTEFLSFMNTELAAFTKNQKDPGVLDRMMKKLDTNSDGQLDFSEFLNLIGGLAMACHDSFLKAVPSQKRT. | 11642.9 | 2 | 105 | S100A11 | 4.26e-14 |
| sp\|P60520\|GBRL2_HUMAN | .MKWMFKEDHSLEHRCVESAKIRAKYPDRVPVIVEKVSGSQIVDIDKRKYLVPSDITVAQFMWIIRKRIQLPSEKAIFLFVDKTVPQSSLTMGQLYEKEKDEDGFLYVAYSGEN(TF)[-147.0602]GF. | 13512.2 | 1 | 117 | GABARAPL2 | 7.55e-11 |
| tr\|D6RF53\|D6RF53_HUMAN | .MQLKPMEINPEMLNKVLSRLGVAGQWRFVDVLGLEEESLGSV(PAPACALLLLFPLTAQ)[-128.0520]. | 6202.23 | 1 | 58 | UCHL1 | 1.15e-07 |
| sp\|Q13404\|UB2V1_HUMAN | M.[Acetyl]-AATTGSGVKVPRNFRLLEELEEGQKGVGDGTVSWGLEDDEDMTLTRWT(GMI)[-0.8795]IGPPRTIYENRIYSLKIECGPKYPEAPPFVRFVTKINMNGVNSSNGVVDPRAISVLAKWQNSYSIKVVLQELRRLMMSKENMKLPQPPEGQCYSN. | 16394.5 | 2 | 147 | UBE2V1 | 1.69e-09 |
| sp\|P63208\|SKP1_HUMAN | M.PSIKLQSSDGEIFEVDVEIAKQSVTIKTMLEDLGMDDEGDDDPV(PL)[+0.0797]PNVNAAILKKVIQWCTHHKDDPPPPEDDENKEKRTDDIPVWDQEFLKVDQGTLFELILAANYLDIKGLLDVTCKTVANMIKGKTPEEIRKTFNIKNDFTEEEEAQVRKENQWCEEK. | 18514.5 | 2 | 163 | SKP1 | 4.53e-13 |
| sp\|P62857\|RS28_HUMAN | .(M)[+42.0624]DTSRVQPIKLARVTKVLGRTGSQGQCTQVRVEFMDDTSRSIIRNVKGPVREGDVLTLLESEREAR.R | 7453.02 | 1 | 66 | RPS28 | 1.77e-07 |
| sp\|P04350\|TBB4A_HUMAN | .MREIVHLQAGQCGNQIGAKFWEVISD(E)[+0.0244]HGIDPTGTYHGDSDLQLERINVYYNEATGGNYVPRA.V | 7031.45 | 1 | 63 | TUBB4A | 3.32e-29 |
| sp\|P61960\|UFM1_HUMAN | M.[Acetyl]-SKVSFKITLTSDPRLPYKVLSVPESTPFTAVLKFAAEEFKVPAATSAIITNDGIGINPAQTA(GNVFLKHGSE)[-346.1060]LRIIPRDRVGSC. | 8676.82 | 2 | 85 | UFM1 | 4.07e-14 |
| sp\|Q99497\|PARK7_HUMAN | M.[Acetyl]-ASKRALVILAKGAEEMETVIPVDVMRRAGIKVTVAGLAGKDPVQCSRDVVICPDASLEDAKKEGPYDVVVLPGGNLGAQNLSESAAVKEILKEQENRKGLIAAICAGPTALLAHEIGFGSKVTTHPLAKDKMMNGGHYTY(SENRVEKDGLILTSRGPGTSFEFAL)[+190.1482]AIVEALNGKEVAAQVKAPLVLKD. | 19980.7 | 2 | 189 | PARK7 | 7.500000000000001e-32 |
| sp\|P68036\|UB2L3_HUMAN | M.[Acetyl]-AASRRLMKELEEIRKCGMKNFRNIQVDEANLLTWQGLIVPDNPPYDKGAFRIEINFPAEYPFKPP(KITFKTKIYHPNIDEKGQVCLPVISAENW)[-1.9024]KPATKTDQVIQSLIALVNDPQPEHPLRADLAEEYSKDRKKFCKNAEEFTKKYGEKRPVD. | 17760.4 | 2 | 154 | UBE2L3 | 2.8e-10 |
| tr\|F8W6P5\|F8W6P5_HUMAN | M.VHLTPEEKSAVTALWGKVNVDEVGGEALGRLLVVYPWTQRFFESFGDLSTPDAVMGNPKVKAHGKKVLGAFSDGLAHLDNLKGTF(ATLS)[-5.6411]. | 9528.36 | 2 | 90 | HBB | 9.27e-08 |
| sp\|P30046\|DOPD_HUMAN | M.PFLELDTNLPANRVPAGLEKRLCAAAASILGKPADRVNVTVRPGLAMALSGSTEPCAQLSISSIGVVGTAEDN(RSHSAHFFEFLTKELALGQDRILIRFFPLESWQI)[-0.9148]GKIGTVMTFL. | 12572.8 | 2 | 118 | DDT | 7.68e-11 |
| sp\|Q96FJ2\|DYL2_HUMAN | M.[Acetyl]-SDRKAVIKNADMSED(MQQDAVDCATQAMEKYNIEKDIAAYIKKEFDKKYNPTWHCIVGRNFGSYVTHETKHFIYFYL)[+197.1801]GQVAILLFKSG. | 10450.0 | 2 | 89 | DYNLL2 | 3e-32 |
| sp\|P68871\|HBB_HUMAN | M.VHLTPEEKSAVTALWGKVNVDEVGGEALGRLLVVYPWTQRFFESFGDLSTPDAVMGNPKVKAHGKK(VLGA)[+400.8905].F | 7944.7 | 2 | 71 | HBB | 1.53e-11 |
| sp\|P30044\|PRDX5_HUMAN | A.PIKVGDAIPAVEVFEGEPGNKVNLAELFKGKKGVLFGVPGAFT(PGCSKTHLPGFVEQAEALKAKGVQVVACLSVNDAFVTGEWGRAHKAEGKVRLLADPTGAFGKETDLLLDDSLVSIFGNRRLKRFSMVVQDGIVKALNVEPDGTGLTCSLAPNIISQL)[+96.0073]. | 16915.0 | 55 | 214 | PRDX5 | 8.17e-18 |
| sp\|P07737\|PROF1_HUMAN | M.[Acetyl]-AGWNAYIDNL(MADGTCQDAAIVGYKDSPSVWAAVPGKTFVNIT)[-1.7482]PAEVGVLVGKDRSSFYVNGLTLGGQKCSVIRDSLLQDGEFSMDLRTKSTGGAPTFNVTVTKTDKTLVLLMGKEGVHGGLINKKCYEMASHLRRS.Q | 14663.7 | 2 | 138 | PFN1 | 2.1e-09 |
| sp\|P63167\|DYL1_HUMAN | M.[Acetyl]-CDRKAVIKNADMSEEMQQDSVECATQALEKYNIEKDIAAHIKKEFDKKYNPTWHCIVGRNFGSYVTHETKHFIYFYLGQVAILLFKSG. | 10271.1 | 2 | 89 | DYNLL1 | 7.45e-12 |
| sp\|P62328\|TYB4_HUMAN | M.[Acetyl]-SDKPDMAEIEKFDKSKLKKTETQEKNPLPSKETIEQEKQAG(ES)[-119.0193]. | 4842.41 | 2 | 44 | TMSB4X | 6.66e-33 |
| sp\|P60903\|S10AA_HUMAN | M.PSQMEHAMETMMFTFHKFAGDKGYLTKEDLRVLMEKEFPGFLENQKDPLAVDKIMKDLDQCRDGKVGFQSFF(S)[+31.0440]LIAGLTIACNDYFVVHMKQKGKK. | 11095.6 | 2 | 97 | S100A10 | 1.13e-11 |
| sp\|Q92686\|NEUG_HUMAN | R.KKIKSGERGRKGPGPGGPGGAG(V)[+97.0369]ARGGAGGGPSGD. | 3183.64 | 44 | 78 | NRGN | 3.13e-15 |
| sp\|P06703\|S10A6_HUMAN | L.(LVAI)[-235.0727]FHKYSGREGDKHTLSKKELKELIQKELTIGSKLQDAEIARLMEDLDRNKDQEVNFQEYVTFLGALALIYNEALKG. | 8825.8 | 12 | 90 | S100A6 | 2.85e-06 |
| sp\|P14174\|MIF_HUMAN | M.PMFIVNTNVPRASVPDGFLSELTQQLAQATGKPPQYIAVHVVPDQL(MA)[+43.4043]FGGSSEPCALCSLHSIGKIGGAQNRSYSKLLCGLLAERLRISPDRVYINYYDMNAANVGWNNSTFA. | 12379.3 | 2 | 115 | MIF | 6.21e-22 |
| sp\|Q96FJ2\|DYL2_HUMAN | M.[Acetyl]-SDRKAVIKNADMS(EDMQQDAVDCATQAMEKYNIEKDIAAYIKKEFDKKYNPTWHCIVGRNFGSYVTHETKHFIYF)[-15.9938]YLGQVAILLFKSG. | 10239.1 | 2 | 89 | DYNLL2 | 3e-32 |
| sp\|P59768\|GBG2_HUMAN | M.[Acetyl]-AS(N)[+1.0229]NTASIAQARKLVEQLKMEANIDRIKVSKAAADLM.A | 4041.21 | 2 | 38 | GNG2 | 2.25e-06 |
| sp\|P14174\|MIF_HUMAN | M.PMFIVNTNVPRASVPDGFLSELTQQLAQATGKPPQYIAVHVVPDQLMAFGGSS(E)[+107.0674]PCALCSLHSIGKIGGAQNRSYSKLLCGLLAERLRISPDRVYINYYDMNAANVGWNNSTFA. | 12444.3 | 2 | 115 | MIF | 6.21e-22 |
| sp\|P56381\|ATP5E_HUMAN | .([Acetyl]-M)[-73.8839]VAYWRQAGLSYIRYSQICAKAVRDALKTEFKANAEKTSGSNVKIVKVKKE. | 5743.12 | 1 | 51 | ATP5F1E | 3.2e-13 |
| sp\|P60903\|S10AA_HUMAN | M.PSQMEHAMETMMFTFHKFAGDKGYLTKEDLRVLMEKEFPGFLENQKDPLAVDKIMKDLDQCRDGKVGFQSFFSLIAGLTI(A)[+16.0076]CNDYFVVHMKQKGKK. | 11081.6 | 2 | 97 | S100A10 | 1.13e-11 |
| sp\|P05204\|HMGN2_HUMAN | M.PKRKAEGDAKGDKAKVKDEPQRRSARLSAKPAPPKPEPKPKKAPAKKGEKVPKGKKGKADAGKEGNNPAENGDAKTDQAQKAEGAGD(AK)[-127.7946]. | 9129.04 | 2 | 90 | HMGN2 | 2.91e-18 |
| sp\|Q92686\|NEUG_HUMAN | K.(KIKSGERGRKGPGPGGPGGA)[+0.0041]GVARGGAGGGPSGD. | 2957.58 | 45 | 78 | NRGN | 5.89e-06 |
| sp\|P37840\|SYUA_HUMAN | M.DVFMKGLSKAKEGVVAA(AEKTKQGVAEAAGKTKEGVLYV)[+173.1102]GSKTKEGVVHGVATVAEKTKEQVTNVGGAVVTGVTAVAQKTVEGAGSIAA.A | 8952.91 | 2 | 90 | SNCA | 7.04e-07 |
| sp\|Q96FJ2\|DYL2_HUMAN | .[Acetyl]-MSDR(KAVIKNADMSEDMQQDAVDCATQAMEKYNIEKDIAAYIKK)[+149.1125]EFDKKYNPTWHCIVGRNFGSYVTHETKHFIYFYLGQVAILLFKSG. | 10535.2 | 1 | 89 | DYNLL2 | 6.22e-11 |
| sp\|P04350\|TBB4A_HUMAN | .MREIVHLQAGQCGNQ(I)[+0.0193]GAKFWEVISDEHGIDPTGTYHGDSDLQLERI.N | 5262.62 | 1 | 47 | TUBB4A | 8.78e-20 |
| sp\|P68871\|HBB_HUMAN | K.(LHV)[-19.2087]DPENFRLLGNVLVCVLAHHFGKEFTPPVQAAYQKVVAGVANALAHKYH. | 5597.65 | 97 | 147 | HBB | 0.001927975 |
| sp\|P0DPH7\|TBA3C_HUMAN | .MRECISIHVGQAGVQIGNACWELYCLEHGIQPDGQM(PSDK)[+41.2992]TIGGGDDSFNTFFSETGAGKHVPRA.V | 7003.38 | 1 | 65 | TUBA3C | 1.58e-22 |
| sp\|P00738\|HPT_HUMAN | D.S(GN)[+214.1995]DVTDIADDGCPKPPEIAHGYVEHSVRYQ.C | 3582.71 | 21 | 51 | HP | 2.46e-09 |
| sp\|Q16143\|SYUB_HUMAN | L.YVGSKTREGVVQGVASVAEKTKEQ(ASH)[+0.0290]LGGAVFSGAGNIA.A | 3959.13 | 39 | 78 | SNCB | 4.62e-07 |
| sp\|Q92686\|NEUG_HUMAN | .[Acetyl]-MDCCTENACSKPDDDIL(D)[-17.9768]IPLDDPGANAAAAKIQASFRGHMAR.K | 4570.11 | 1 | 43 | NRGN | 4.17e-18 |
| sp\|P62937\|PPIA_HUMAN | .(MVN)[-129.9831]PTVFFDIAVDGEPLGRVSFELFADKVPKTAENFRAL.S | 4208.29 | 1 | 39 | PPIA | 3.62e-14 |
| sp\|P60709\|ACTB_HUMAN | .(MDDD)[-87.8588]IAALVVDNGSGMCKAGFAGDDAPRAVFPSIVGRPRHQG.V | 4223.12 | 1 | 42 | ACTB | 5.82e-05 |

# **Table S3.** Proteoforms exclusively detected in healthy control samples via SEC-CZE-MS/MS.

| Protein accession | Proteoform | Precursor mass | First residue | Last residue | Gene | E-value |
| --- | --- | --- | --- | --- | --- | --- |
| sp\|P0DP23\|CALM1_HUMAN | M.[Acetyl]-ADQLTEEQI(AEFKEAFSLFDKDGDGTITTKELGTVMRSLGQNPTEAELQDMINEVDADGNGTIDFPEFLTMMARKMKDTDSEEEIREAFRVFDKDGNGYISAAELRHVMTNLGEKLTDEEVDEMIREADIDGDGQV)[+157.3898]NYEEFVQMMTAK. | 16893.9 | 2 | 149 | CALM1 | 1.2e-19 |
| sp\|P0DP23\|CALM1_HUMAN | M.[Acetyl]-ADQLTEEQIAEFKEAFSLFDKDGDGTITTKELGTVMRSLGQNPTEAELQDMINEVDADGNGTIDFPEFLTMMARKMKDTDSEEEIREAFRVF(D)[+103.0277]KDGNGYISAAELRHVMTNLGEKLTDEEVDEMIREADIDGDGQVNYEEFVQMMTAK. | 16839.9 | 2 | 149 | CALM1 | 1.2e-19 |
| sp\|P0DP23\|CALM1_HUMAN | M.[Acetyl]-ADQLTEEQIAEFKEAFSLFDKDGDGTITTKELGTVMRSLGQNPTEAELQDMINEVDADGNG(TIDFPEFLTMMARKMKDTDSEEEIREAFRVFD)[+137.0388]KDGNGYISAAELRHVMTNLGEKLTDEEVDEMIREADIDGDGQVNYEEFVQMMTAK. | 16874.9 | 2 | 149 | CALM1 | 1.2e-19 |
| sp\|P61604\|CH10_HUMAN | M.[Acetyl]-AGQAFRKFLPLFDRVLVERSAAETVTKGGIML(PEKSQGKVLQAT)[+56.0131]VVAVGSGSKGKGGEIQPVSVKVGDKVLLPEYGGTKVVLDDKDYFLFRDGDILGKYVD. | 10892.9 | 2 | 102 | HSPE1 | 3.0500000000000003e-32 |
| sp\|P0CG47\|UBB_HUMAN | .MQIFVKTLTGKTITLEVE(PSDTIENVKAKIQD)[+111.8832]KEGIPPDQQRLIFAGKQLEDGRTLSDYNIQKESTLHLVLRLRGG.M | 8672.33 | 1 | 76 | UBB | 1.6200000000000002e-21 |
| sp\|Q16143\|SYUB_HUMAN | .[Acetyl]-MDVFMKGLSMAKEGVVAAAEKTKQGVTEAAEKTKEGVLYVGSKTREGVVQGVASVAEKTKEQASHLGGAVFSGAGNIAAATGLVKREEFPTDLKPEEVAQEAAEEPL(IEPLMEPEGESYEDPPQEEYQEYE)[+117.2621]PEA. | 14437.0 | 1 | 134 | SNCB | 2.6999999999999997e-25 |
| sp\|P37840\|SYUA_HUMAN | .[Acetyl]-MDVFMKGLSKAKEGVVAAAEKTKQGVAEAAGKTKEGVLYVGSKTKEGVVHGVATVAEKTKEQVTNVGGAVVTGVTAVAQKTVEGAGSIAAATGFVKKDQLGKNEEGAPQEGILEDMPVDPDNEAYE(M)[+20.1565]PSEEGYQDYEPEA. | 14514.4 | 1 | 140 | SNCA | 2.61e-27 |
| sp\|P0CG47\|UBB_HUMAN | .MQIFVKTLTGKTITLEVE(PSD)[+60.9725]TIENVKAKIQDKEGIPPDQQRLIFAGKQLEDGRTLSDYNIQKESTLHLVLRLRGG.M | 8619.62 | 1 | 76 | UBB | 1.6200000000000002e-21 |
| sp\|P0CG47\|UBB_HUMAN | .MQIFVKTLTGKTITLEVE(PSDTIENVKAKIQDK)[+167.9569]EGIPPDQQRLIFAGKQLEDGRTLSDYNIQKESTLHLVLRLR.G | 8614.41 | 1 | 74 | UBB | 2.1e-20 |
| sp\|P30049\|ATPD_HUMAN | Y.AEAAAAPAAASGPNQMSFTFA(S)[+38.0288]PTQVFFNGANVRQVDVPTLTGAFGILAAHVPTLQVLRPGLVVVHAEDGTTSKYFVSSGSIAVNADSSVQLLAEEAVTLDMLDLGAAKANLEKAQAELVGTADEATRAEIQIRIEANEALVKALE. | 15048.8 | 23 | 168 | ATP5F1D | 9.26e-23 |
| sp\|O14810\|CPLX1_HUMAN | .[Acetyl]-MEFVMKQALGGATKDMGKMLGGDEEKDPDAAKKEEERQEALRQAEEERKAKYAKMEAEREAVRQGIRDKYGIKK(KEEREAE)[-0.8970]AQAAMEANSEGSLTRPKKAIPPGCGDEVEEEDESILDTVIKYLPGPLQDMLKK. | 15061.8 | 1 | 134 | CPLX1 | 1.28e-14 |
| sp\|P0CG47\|UBB_HUMAN | .MQIFVKTLTGKTITLEVE(PSD)[+116.8904]TIENVKAKIQDKEGIPPDQQRLIFAGKQLEDGRTLSDYNIQKESTLHLVLRLRGG.M | 8675.5 | 1 | 76 | UBB | 1.6200000000000002e-21 |
| sp\|P30049\|ATPD_HUMAN | Methyl]AAHVPTLQVLRPGLVVVHAEDGTTSKYFVSSGSIAVNADSSVQLLAEEAVTLDMLDLGAAKANLEKAQAELVGTADEATRAEIQIRIEANEALVKALE.;Y.AEAAAAPAA(ASGPNQMSFTFASPTQVFFNGANVRQVDVPTLTGAFGIL)[Phospho | 15104.8 | 23 | 168 | ATP5F1D | 9.26e-23 |
| sp\|Q16143\|SYUB_HUMAN | .[Acetyl]-MDVFMKGLSMAKEGVVAAAEKTKQGVTEAAEKTKEGVLYVGSKTREGVVQGVASVAEKTKEQASHLGGAVFSGAGNIAAATGLVKREEFPTDLKPEEVAQEAAEEPLIEPLMEPEGESYEDP(PQEEYQEYE)[+135.0175]PEA. | 14455.0 | 1 | 134 | SNCB | 2.6999999999999997e-25 |
| sp\|P0CG47\|UBB_HUMAN | .MQIFVKTLTGKTITLE(VEPSDTIENVKAKIQDK)[+150.8585]EGIPPDQQRLIFAGKQLEDGRTLSDYNIQKESTLHLVLRLR.G | 8597.44 | 1 | 74 | UBB | 2.1e-20 |
| sp\|P62328\|TYB4_HUMAN | M.[Acetyl]-SDKPDMAEIEKFDKSKLKKTETQEKN(PLPSKETIEQE)[+168.8179]KQAGES. | 5129.33 | 2 | 44 | TMSB4X | 1.37e-34 |
| sp\|P62328\|TYB4_HUMAN | M.[Acetyl]-SDKPDMAEIEKFDKSKLKK(TETQEK)[+61.9560]NPLPSKETIEQEKQAGES. | 5021.45 | 2 | 44 | TMSB4X | 1.37e-34 |
| sp\|Q16143\|SYUB_HUMAN | M.AKEGVVAAAEKTKQGVTEAAEKTKEGVLYVGSKTREGVVQGVASVAEKTKEQASHLGGAVFSGAGNIAAATGLVKREEFPTDLKPEEVAQEAAEEPLIEPLME(PEGESYED)[+57.0198]PPQEEYQEYEPEA. | 13196.7 | 11 | 134 | SNCB | 8.39e-13 |
| sp\|P63313\|TYB10_HUMAN | M.[Acetyl]-ADKPDMGEIASFDKAKLKKTETQEKN(TL)[+55.9513]PTKETIEQEKRSE.I | 4789.41 | 2 | 42 | TMSB10 | 4.46e-26 |
| sp\|P62328\|TYB4_HUMAN | M.[Acetyl]-SDKPDMAEIEKFDKSKLKKTETQE(K)[+55.9475]NPLPSKETIEQEKQ.A | 4673.35 | 2 | 40 | TMSB4X | 1.5399999999999999e-24 |
| sp\|P62942\|FKB1A_HUMAN | M.GVQVETIS(PGDGRTFPKRGQTCVVHYTGMLED)[+304.1513]GKKFDSSRDRNKPFKFMLGKQEVIRGWEEGVAQMSVGQRAKLTISPDYAYGATGHPGIIPPHATLVFDVELLKLE. | 12117.4 | 2 | 108 | FKBP1A | 1.22e-11 |
| sp\|P62328\|TYB4_HUMAN | M.[Acetyl]-SDKPDMAEIEKFDKSKLKKTETQEK(N)[+112.8712]PLPSKETIEQEKQAG.E | 4857.32 | 2 | 42 | TMSB4X | 1e-22 |
| sp\|Q96FJ2\|DYL2_HUMAN | M.[Acetyl]-SDRKAVIKNAD(MSEDMQQDAVDCATQAMEKYNIEKDIAAYIKKEFDKKYNPTWHCIVGRNFGSYVTHETKHFIYFYL)[-0.9436]GQVAILLFKSG. | 10252.2 | 2 | 89 | DYNLL2 | 1.25e-15 |
| sp\|O75368\|SH3L1_HUMAN | M.VIRVYIASSSGSTAIKKKQQDVLGFLEANKIGFEEKDIAANEENRKWMRENVPENSRPATGYPLPPQIFNESQYRGDYDAFFEARENNAVYAFLGL(T)[+37.0813]APPGSKEAEVQAKQQA. | 12672.5 | 2 | 114 | SH3BGRL | 1.2e-16 |
| sp\|P30049\|ATPD_HUMAN | Y.AEAAAAPAAASGPNQMSFT(F)[+22.0540]ASPTQVFFNGANVRQVDVPTLTGAFGILAAHVPTLQVLRPGLVVVHAEDGTTSKYFVSSGSIAVNADSSVQLLAEEAVTLDMLDLGAAKANLEKAQAELVGTADEATRAEIQIRIEANEALVKALE. | 15032.9 | 23 | 168 | ATP5F1D | 9.26e-23 |
| sp\|P62328\|TYB4_HUMAN | M.[Acetyl]-SDKPDMAEIEKFDKSKLKKTETQE(K)[+113.8764]NPLPSKETIEQEKQAGES. | 5075.37 | 2 | 44 | TMSB4X | 1.37e-34 |
| sp\|P30086\|PEBP1_HUMAN | M.PVDLSKWSGPLSLQEVDEQPQHPLHVTYAGAAVDELGKVLTPTQVKNRPTSISWDGLDSGKLYTLVLTDPDAPSRKDPKYREWHHFLVVNMKGNDISSGTVLSDYVGSGPPKGTGLHRYVWLVYEQD(RPLKCD)[+119.0865]EPILSNRSGDHRGKFKVASFRKKYELRAPVAGTCYQAEWDDYVPKLYEQLSGK. | 21030.9 | 2 | 187 | PEBP1 | 1.45e-14 |
| sp\|P07196\|NFL_HUMAN | K.DEP(PSEGEAEEEEKDKEEAEEE)[+55.9514]EAAEEEEAAKEESEEAKEEEEGGEGEEGEETKEAEEEEKKVEGAGEEQAAKKKD. | 8481.58 | 468 | 543 | NEFL | 5.72e-15 |
| sp\|P63313\|TYB10_HUMAN | M.[Acetyl]-ADKPDMGEIASFDKAKLKKTETQEKN(TL)[+112.8821]PTKETIEQEKRSEIS. | 5047.43 | 2 | 44 | TMSB10 | 1.9000000000000002e-29 |
| sp\|P30049\|ATPD_HUMAN | Y.AEAAAAPAAASGPNQMSFTFASPTQV(FFNGANVRQVDVPTLTGAFGILAAH)[+113.8849]VPTLQVLRPGLVVVHAEDGTTSKYFVSSGSIAVNADSSVQLLAEEAVTLDMLDLGAAKANLEKAQAELVGTADEATRAEIQIRIEANEALVKALE. | 15124.7 | 23 | 168 | ATP5F1D | 9.26e-23 |
| sp\|P62937\|PPIA_HUMAN | M.VNPTVFFDIAVDGE(PLGRVSFELFADKVPKTAENFRALSTGEKGFGYKGSCFHRIIPGFMCQGGDFTRHNGTGGKSIYGEKFEDENFILKHTGPGILSMANAGPNTNGSQFFICTAKTEWLDGKHVVFGKVKEGMNIVEAMERFGSRNGK)[-2.8843]TSKKITIADCGQLE. | 17867.2 | 2 | 165 | PPIA | 7.69e-07 |
| sp\|Q5VTU8\|AT5EL_HUMAN | M.VAYWRQAGLSYIRYSQI(CAKVVRDALKTEFKANAK)[-27.0500]KTSGNSVKIVKVKKE. | 5645.18 | 2 | 51 | ATP5F1EP2 | 9.21e-12 |
| sp\|P30049\|ATPD_HUMAN | Y.AEAAAAPAAASGPNQMSFTFAS(PTQVFFNGANVRQVDVPTLTGAFGILAAHVPTLQVLRPGLVVVHAEDGTTSKYFVSSGSI)[+78.0189]AVNADSSVQLLAEEAVTLDMLDLGAAKANLEKAQAELVGTADEATRAEIQIRIEANEALVKALE. | 15087.8 | 23 | 168 | ATP5F1D | 9.26e-23 |
| sp\|P43004\|EAA2_HUMAN | Y.AAHNSVIVDE(CKVTLAANGKSADC)[-1.0114]SVEEEPWKREK. | 3811.9 | 540 | 574 | SLC1A2 | 4.4e-15 |
| sp\|Q6U841\|S4A10_HUMAN | .[Acetyl]-MEIKDQGAQMEP(L)[+0.0411]LPTRNDEEAVVDRGGTRSILKTHFEKEDLEGHRT.L | 5417.79 | 1 | 47 | SLC4A10 | 7.43e-06 |
| sp\|P30049\|ATPD_HUMAN | Oxidation]AVNADSSVQLLAEEAVTLDMLDLGAAKANLEKAQAELVGTADEATRAEIQIRIEANEALVKALE.;Y.AEAAAAPAAASGPNQMSFTFAS(PTQVFFNGANVRQVDVPTLTGAFGILAAHVPTLQVLRPGLVVVHAEDGTTSKYFVSSGSI)[Phospho | 15106.8 | 23 | 168 | ATP5F1D | 9.26e-23 |
| sp\|Q92686\|NEUG_HUMAN | R.KKIKSGERGRKGPGPGGPGGAGVARGGAGGG(PS)[+55.9297]GD. | 3141.58 | 44 | 78 | NRGN | 1.23e-14 |
| sp\|O75368\|SH3L1_HUMAN | M.VIRVYIASSSGSTAIKKKQQDVLGFLEANKIGFEEKDIAANEENRKWMRENVPENSRPATGYPLPPQIFNESQYRGDYDAFFEAR(ENNAVYA)[+182.1290]FLGLTAPPGSKEAEVQAKQQA. | 12818.6 | 2 | 114 | SH3BGRL | 1.2e-16 |
| tr\|E9PPW2\|E9PPW2_HUMAN | M.GSWALLWPPLLFTGLLVRPPGTMAQAQYCSVNKDIFEVEENTNVTEPLVDIHVPEGQEVTLGALSTPFAFRI(QGNQLFLNVTPDYEEKSLLEAQLLCQSGG)[-398.2381]TLTKEIRVEEDTKVNSTVIPETQLQAEDRDKDDILFYTLQEMTAGASDYFSLVSVNRPALRLD. | 17866.2 | 2 | 165 | CDHR5 | 0.000294579 |
| sp\|P63313\|TYB10_HUMAN | M.(A)[+38.1743]DKPDMGEIASFDKAKLKKTETQEKNTLPTKETIEQEKRSEIS. | 4928.59 | 2 | 44 | TMSB10 | 1.9000000000000002e-29 |
| sp\|P62942\|FKB1A_HUMAN | M.GVQVETIS(PGD)[+120.0990]GRTFPKRGQTCVVHYTGMLEDGKKFDSSRDRNKPFKFMLGKQEVIRGWEEGVAQMSVGQRAKLTISPDYAYGATGHPGIIPPHATLVFDVELLKLE. | 11932.3 | 2 | 108 | FKBP1A | 1.22e-11 |
| sp\|P0DP23\|CALM1_HUMAN | M.[Acetyl]-ADQLTEEQIAEFKEAFSLFDKDGDGTITTKELGTVMRSLGQNPTEAELQDMINEVDADGNG(TIDFPEFLTMMARKMKDTDSEEEIREAFRV)[+82.0761]FDKDGNGYISAAELRHVMTNLGEKLTDEEVDEMIREADIDGDGQVNYEEFVQMMTAK. | 16821.0 | 2 | 149 | CALM1 | 1.2e-19 |
| sp\|P55087\|AQP4_HUMAN | Q.TKGSYMEVEDNRSQVETDDLIL(K)[+0.0454]PGVVHVIDVDRGEEKKGKDQSGEVLS.S | 5427.83 | 273 | 321 | AQP4 | 7.02e-12 |
| sp\|P30049\|ATPD_HUMAN | Y.AEAAAAPAAASGPNQMSFT(F)[+20.0324]ASPTQVFFNGANVRQVDVPTLTGAFGILAAHVPTLQVLRPGLVVVHAEDGTTSKYFVSSGSIAVNADSSVQLLAEEAVTLDMLDLGAAKANLEKAQAELVGTADEATRAEIQIRIEANEALVKALE. | 15029.9 | 23 | 168 | ATP5F1D | 9.26e-23 |
| sp\|Q16352\|AINX_HUMAN | S.STGLSLKKE(EE)[+0.0369]EEEASKVASKKTSQIGESFEEILEETVISTKK.T | 4754.53 | 441 | 483 | INA | 4.27e-22 |
| sp\|P62942\|FKB1A_HUMAN | M.GVQVETIS(PGDGRTFPKRGQTCVVHYTGMLEDG)[+195.1031]KKFDSSRDRNKPFKFMLGKQEVIRGWEEGVAQMSVGQRAKLTISPDYAYGATGHPGIIPPHATLVFDVELLKLE. | 12006.3 | 2 | 108 | FKBP1A | 1.22e-11 |
| sp\|P11137\|MTAP2_HUMAN | G.(QE)[+1.0508]PILTEKETELKLEEKTTISDKEAVPKESKPPKPADEEIGIIQTSTEHTFSEQKDQEPTTDMLKQDSFPVSLEQAVTDSAMTSKTLEK.A | 9986.2 | 431 | 519 | MAP2 | 6.18e-08 |
| sp\|P10412\|H14_HUMAN | M.[Acetyl]-SETAPAAPAAPAPAEKT(PVKKKARKSAGAAKRK)[+243.1906]ASGPPVSELITKAVAASKER.S | 5561.26 | 2 | 54 | H1 | 5.08e-05 |
| sp\|P62328\|TYB4_HUMAN | M.[Acetyl]-SDKPDMAEIEKFDKSKLKKTETQEK(N)[+0.0294]PLPSKETIE.Q | 4103.2 | 2 | 36 | TMSB4X | 9.4e-08 |
| sp\|P63313\|TYB10_HUMAN | M.[Acetyl]-ADKPDM(GE)[+0.0268]IASFDKAKLKKTETQEKN.T | 2963.57 | 2 | 27 | TMSB10 | 3.04e-06 |
| sp\|P81605\|DCD_HUMAN | A.YDPEAASAPGS(GNPC)[+305.0996]HEASAAQKENAGEDPGLARQAPKPRKQ.R | 4605.21 | 20 | 61 | DCD | 9.63e-22 |
| tr\|G4Y816\|G4Y816_HUMAN | .MDVFMKGLSMAKEGVVAAAEKTKQGVTEAAEKTKEGVLYVVAEKTKE(QASHLGGAVFSGAGNIAAATGLVKREEFPTDLKPEEV)[+434.2830]AQEAAEEPLIEPLMEPEGESYEDPPQEEYQEYEPEA. | 13357.8 | 1 | 120 | SNCB | 1.29e-13 |
| sp\|P05387\|RLA2_HUMAN | .MRYVASYLLAALGGNSSPSAKDIKKILDSVGIEADDDRLNKVISELNGKNIEDVIAQGIGKLASVPAGGAV(A)[+55.0194]VSAAPGSAAPAAGSAPAAAEEKKDEKKEESEESDDDMGFGLFD. | 11714.0 | 1 | 115 | RPLP2 | 4.28e-21 |
| sp\|P55087\|AQP4_HUMAN | T.KGSYMEVEDNRSQVETDDLIL(K)[+0.0250]PGVVHVIDVDRGEEKKGKDQSGEVLSSV. | 5512.86 | 274 | 323 | AQP4 | 3.54e-10 |
| sp\|P07197\|NFM_HUMAN | K.(V)[+0.0190]EAPKLKVQHKFVEEIIEETKVEDEK.S | 3093.73 | 441 | 466 | NEFM | 7.28e-09 |
| sp\|P11137\|MTAP2_HUMAN | L.VASLEDMKQKTEPSLVVPGIDLPKEPPTPKEQKDWFIEMPTEAKKDEWGLVAPISPGPLTPMREKDVFDDIPKWEGKQFDSPMPSPFQGGSFT(L)[-0.9274]PLDVMKNEIVTETSPFAPA.F | 12571.6 | 231 | 343 | MAP2 | 6.62e-09 |
| sp\|P55087\|AQP4_HUMAN | K.PGVVHVIDVDRGEEKKGKDQSGE(V)[+241.1840]LSSV. | 3203.76 | 296 | 323 | AQP4 | 2.39e-06 |
| sp\|P10412\|H14_HUMAN | .(MSET)[-90.9449]APAAPAAPAPAEKTPVKKKARKSAGAAKRKASGPPVSELI.T | 4275.5 | 1 | 44 | H1 | 8.39e-07 |
| sp\|P10606\|COX5B_HUMAN | V.PSI(S)[-3.9759]NKRIVGCICEEDNTSVVWFWLHKGEAQRCPRCGAHYKLVPQQLAH. | 5598.84 | 81 | 129 | COX5B | 8.7e-05 |
| sp\|O76070\|SYUG_HUMAN | .[Acetyl]-MDVFK(K)[+0.0320]GFSIAKEGVVGAVEKTKQGVTEAAEKTKEG.V | 3838.12 | 1 | 36 | SNCG | 7.18e-16 |
| sp\|P36578\|RL4_HUMAN | C.(SALA)[+0.2115]ASALPALVMSKGHRIEEVPELPLVVEDKVEGYKKTKEAV.L | 4601.64 | 126 | 168 | RPL4 | 1.52e-09 |
| tr\|A0A7P0T936\|A0A7P0T936_HUMAN | .[Acetyl]-MAEPR(QEFEVMEDHA)[-131.0002]GTYGLGDRKDQGGYTMHQDQEGDTDAGLKAEEAGIGDTPSLEDEAAGHVTQARM.V | 7358.42 | 1 | 69 | MAPT | 8.03e-11 |
| sp\|P12277\|KCRB_HUMAN | M.PFSNSHNALKLRFPAEDE(F)[+0.0386]PDLSAHNNHMAKVLTPE.L | 4073.09 | 2 | 37 | CKB | 8.57e-09 |
| sp\|P63313\|TYB10_HUMAN | M.GEIASFDKAK(LK)[+0.0344]KTETQEKNTLPTKETIEQEKRSE.I | 4034.19 | 8 | 42 | TMSB10 | 1.65e-13 |
| sp\|Q9NZR1\|TMOD2_HUMAN | M.[Acetyl]-ALPFQKELEKYKNIDEDELLGKLSEEELKQLENVLDD(L)[+1.1100]DPESAMLPAGFRQKDQTQKAATGPFDREHLL.M | 7965.21 | 2 | 70 | TMOD2 | 2.35e-07 |
| sp\|P10636\|TAU_HUMAN | K.KIETHKLTFRENAKAKTDHGAEIVYKSPVVSGDTSPRHLSNVSSTG(S)[+0.0478]IDMVDSPQLATLADEVSASLAKQGL. | 7631.09 | 687 | 758 | MAPT | 1.15e-13 |
| sp\|P63313\|TYB10_HUMAN | M.[Acetyl]-ADKPDMGE(IA)[+0.0307]SFDKAKLKKTETQEKNTLPTKETIE.Q | 3975.11 | 2 | 36 | TMSB10 | 1.11e-08 |
| sp\|O15240\|VGF_HUMAN | K.NAPPEPVPPPRAAPAPTHVRSPQPP(P)[-0.9453]PAPAPARDELPDWNEVLPPWDREEDEVYPPGPYHPFPNYIRP.R | 7592.91 | 485 | 552 | VGF | 2.71e-09 |
| sp\|P36542\|ATPG_HUMAN | M.ATLKDITRRLKSIKNIQKITKSMK(M)[+0.0190]VA.A | 3114.92 | 26 | 52 | ATP5F1C | 8.42e-07 |
| sp\|P62328\|TYB4_HUMAN | .(MSDK)[-82.7960]PDMAEIEKFDKSKLKKTETQEKNPLPSKETIEQEK.Q | 4493.44 | 1 | 39 | TMSB4X | 3.01e-11 |
| sp\|P63313\|TYB10_HUMAN | I.ASFDKAKLKKTETQEKNTLPTKETIEQEKRS(EIS)[-200.0897]. | 3735.06 | 11 | 44 | TMSB10 | 1.12e-17 |
| sp\|P63313\|TYB10_HUMAN | M.[Acetyl]-ADKPDMGEIA(SFDKAKLKKTE)[+183.0584]TQEKNTLPTKETIEQEKRSEIS. | 5116.63 | 2 | 44 | TMSB10 | 1.9000000000000002e-29 |
| sp\|P62328\|TYB4_HUMAN | E.KFDKSKLKKTETQEKNPLPSKET(IE)[+0.0208]QEKQAGES. | 3803.08 | 12 | 44 | TMSB4X | 3.67e-08 |
| sp\|O76070\|SYUG_HUMAN | T.VATKTVEEAE(N)[+0.0068]IAVTSGVVRKEDLRPSAPQQEGEASKEKEEVAEEAQSGGD. | 5380.72 | 77 | 127 | SNCG | 1.6e-20 |
| sp\|Q8N111\|CEND_HUMAN | .[Acetyl]-MESRGKSASSPKPDTKVPQVTT(E)[+0.0464]AKVPPAADGKAPLTKPSKKEAPAEK.Q | 5011.8 | 1 | 48 | CEND1 | 3.04e-13 |
| tr\|J3KRX5\|J3KRX5_HUMAN | C.HIEMILTEKEQIVPKPEEEVAQKK(KISQK)[-128.0664]K. | 3429.98 | 145 | 174 | RPL17 | 1.53e-09 |
| sp\|O43237\|DC1L2_HUMAN | L.SKKTGSPGSPGAGGVQSTAKKSGQKTVLSNVQEELDRMTRKPDSMVTNSS(TE)[+0.0381]NEA. | 5691.94 | 438 | 492 | DYNC1LI2 | 3.61e-07 |
| sp\|Q7L0J3\|SV2A_HUMAN | .[Acetyl]-MEE(GFRDR)[+0.0330]AAFIRGAKDIAKEVKKHAAK.K | 3213.8 | 1 | 28 | SV2A | 7.41e-10 |
| sp\|O76070\|SYUG_HUMAN | E.KTKEQANAVSEAVVSSVNTVATKTVEEAENIAV(TSGVVRKEDLRPSAPQQEGEASKEKEEVAEEA)[+55.9217]QSGGD. | 7380.67 | 58 | 127 | SNCG | 2.68e-31 |
| sp\|P56211\|ARP19_HUMAN | K.AKMKNKQLPTAAPDKTEVTGDH(I)[+0.0297]PTPQDLPQRKPSLVASKLAG. | 4576.59 | 70 | 112 | ARPP19 | 5.95e-15 |
| sp\|P07196\|NFL_HUMAN | M.STRSFPSYYTSHVQEE(Q)[+0.0011]IEVEETIEAAKAEEAK.D | 3785.86 | 435 | 467 | NEFL | 8.24e-15 |
| sp\|P12277\|KCRB_HUMAN | M.PFSNSHNALKLRFPAEDE(F)[+0.0323]PDLSAHNNHMA.K | 3405.69 | 2 | 31 | CKB | 5.55e-10 |
| sp\|Q7L1I2\|SV2B_HUMAN | .[Acetyl]-MDDYKYQDNYGGYAPSD(GYYRGNESN)[+1.0094]PEEDAQSDVTEGHDEEDEIYEGEYQGIPHPDDVKAKQAKMAPSR.M | 8004.51 | 1 | 70 | SV2B | 4.99e-16 |
| sp\|P21579\|SYT1_HUMAN | T.(GA)[+0.0240]ELRHWSDMLANPRRPIAQWHTLQVEEEVDAMLAVKK. | 4424.35 | 385 | 422 | SYT1 | 6.4e-08 |
| sp\|O95674\|CDS2_HUMAN | M.[Acetyl]-TELRQRVAHEPVAPPEDKESESEAKVDGETASDSESRA(E)[+0.0354]SAPLPVS.A | 4958.48 | 2 | 47 | CDS2 | 5.86e-12 |
| sp\|Q16555\|DPYL2_HUMAN | S.SAKTSPAKQQAPPVRNLHQSGFSLSGA(Q)[+55.9481]IDDNIPR.R | 3771.93 | 518 | 552 | DPYSL2 | 4.6e-12 |
| sp\|O43768\|ENSA_HUMAN | K.MKNKQLPSAGPDKNLVTGDH(I)[+0.0280]PTPQDLPQRKSSLVTSKLAGGQVE. | 4779.63 | 77 | 121 | ENSA | 0.000837995 |
| sp\|P56211\|ARP19_HUMAN | K.MKNKQLPTAAPDKTEVTGDH(I)[+0.0352]PTPQDLPQRKPSLVASKLAG. | 4377.45 | 72 | 112 | ARPP19 | 4.43e-08 |
| tr\|A6NMQ3\|A6NMQ3_HUMAN | K.NKQLPSAGPDKNLVTGDH(I)[+0.0292]PTPQDLPQRKSSLVTSKLAG. | 4107.3 | 102 | 140 | ENSA | 1.02e-11 |
| sp\|P12277\|KCRB_HUMAN | F.DPIIEDRHGGYKPSDEHKTDLNPDNLQGGDDLDPNY(VL)[-174.9882]SS.R | 4261.09 | 90 | 129 | CKB | 1.51e-05 |
| sp\|P10636\|TAU_HUMAN | M.[Acetyl]-AEPRQE(F)[+0.0258]EVMEDHAGTYGLGDRKDQGGYTMHQDQEGDTDAGLK.E | 4822.2 | 2 | 44 | MAPT | 4.08e-12 |
